# ERM-1 Phosphorylation and NRFL-1 Redundantly Control Lumen Formation in the *C. elegans* Intestine

**DOI:** 10.3389/fcell.2022.769862

**Published:** 2022-02-07

**Authors:** Jorian J. Sepers, João J. Ramalho, Jason R. Kroll, Ruben Schmidt, Mike Boxem

**Affiliations:** ^1^ Division of Developmental Biology, Department of Biology, Faculty of Science, Institute of Biodynamics and Biocomplexity, Utrecht University, Utrecht, Netherlands; ^2^ Laboratory of Biochemistry, Wageningen University and Research, Wageningen, Netherlands

**Keywords:** ezrin, radixin, moesin, ERM-1, NHeRF, EBP50, E3KARP, NRFL-1

## Abstract

Reorganization of the plasma membrane and underlying actin cytoskeleton into specialized domains is essential for the functioning of most polarized cells in animals. Proteins of the ezrin-radixin-moesin (ERM) and Na^+^/H^+^ exchanger 3 regulating factor (NHERF) family are conserved regulators of cortical specialization. ERM proteins function as membrane-actin linkers and as molecular scaffolds that organize the distribution of proteins at the membrane. NHERF proteins are PDZ-domain containing adapters that can bind to ERM proteins and extend their scaffolding capability. Here, we investigate how ERM and NHERF proteins function in regulating intestinal lumen formation in the nematode *Caenorhabditis elegans*. *C. elegans* has single ERM and NHERF family proteins, termed ERM-1 and NRFL-1, and ERM-1 was previously shown to be critical for intestinal lumen formation. Using CRISPR/Cas9-generated *nrfl-1* alleles we demonstrate that NRFL-1 localizes at the intestinal microvilli, and that this localization is depended on an interaction with ERM-1. However, *nrfl-1* loss of function mutants are viable and do not show defects in intestinal development. Interestingly, combining *nrfl-1* loss with *erm-1* mutants that either block or mimic phosphorylation of a regulatory C-terminal threonine causes severe defects in intestinal lumen formation. These defects are not observed in the phosphorylation mutants alone, and resemble the effects of strong *erm-1* loss of function. The loss of NRFL-1 did not affect the localization or activity of ERM-1. Together, these data indicate that ERM-1 and NRFL-1 function together in intestinal lumen formation in *C. elegans*. We postulate that the functioning of ERM-1 in this tissue involves actin-binding activities that are regulated by the C-terminal threonine residue and the organization of apical domain composition through NRFL-1.

## Introduction

The establishment of molecularly and functionally distinct apical, basal, and lateral domains is a key feature of polarized epithelial cells. The outside-facing apical domain has a different lipid and protein composition than the basal and lateral domains and is often decorated by microvilli. The specialization of the apical domain and microvilli formation requires the activities of the ezrin/radixin/moesin (ERM) family of proteins. ERM proteins consist of an N-terminal band Four-point-one/ezrin/radixin/moesin (FERM) domain that mediates binding to the plasma membrane and membrane-associated proteins, a C-terminal tail that mediates actin binding, and a central α-helical linker region (Fehon et al., 2010; McClatchey, 2014). In the cytoplasm, ERM proteins are kept in an inactive, closed, conformation that masks most of regulatory and protein interaction motifs due to an intramolecular interaction between the N- and C-terminal domains (Gary and Bretscher, 1995; Magendantz et al., 1995; Pearson et al., 2000; Li et al., 2007). Binding to the plasma membrane lipid phosphatidylinositol-(4,5) bisphosphate (PIP_2_) as well as phosphorylation of a conserved C-terminal threonine residue (T567 in ezrin) promote the transition to an open and active conformation that can link the plasma membrane to the underlying actin cytoskeleton and control the spatial distribution of protein complexes at the membrane (Simons et al., 1998; Nakamura et al., 1999; Barret et al., 2000; Coscoy et al., 2002; Yonemura et al., 2002; Fievet et al., 2004; Hao et al., 2009; Roch et al., 2010).

The ability of ERM proteins to associate with other proteins can be extended by binding to the scaffolding proteins NHERF1 and NHERF2 (Na^+^/H^+^ exchanger regulatory factors 1 and 2). NHERF1/2 were identified as co-regulators of the Na^+^/H^+^ exchanger NHE3 in kidney epithelial cells (Weinman et al., 1993; Yun et al., 1997; Lamprecht et al., 1998). Independently, NHERF1 was identified as the ERM-binding phosphoprotein 50 (EBP50), based on its ability to interact with activated ezrin and moesin (Reczek et al., 1997). NHERF1/2 are closely related proteins that contain two postsynaptic density 95/disks large/zona occludens-1 (PDZ) domains and an ERM-binding (EB) C-terminal tail that can bind to the FERM domain of active ERM proteins. Since their discovery, a large variety of NHERF1/2 interactors have been identified, including transporters like the cystic fibrosis transmembrane conductance regulator (CFTR) (Seidler et al., 2009), growth factor receptors including EGFR and PDGFR (Maudsley et al., 2000; Lazar et al., 2004), and other scaffold proteins such as the NHERF family member PDZK1 (PDZ domain containing 1) (LaLonde and Bretscher, 2009).

The functional significance of the interaction of NHERF1/2 with ERM proteins is best understood for NHERF1/EBP50. In JEG3 cells, NHERF1/EBP50 promotes microvilli formation or stability by acting as a linker between ezrin and PDZK1, and mice lacking either ezrin or NHERF1/EBP50 show similar defects in microvilli formation and organization in the intestine (Morales et al., 2004; Saotome et al., 2004; Garbett et al., 2010; LaLonde et al., 2010). In a model of MDCK cells developing into 3D cysts, a complex of NHERF1/EBP50, ezrin, and Podocalyxin promotes apical identity and is required for lumen formation (Bryant et al., 2014). In a different 3D cyst model grown from Caco-2 colorectal cells, NHERF1/EBP50 is similarly required for apical–basal polarization and lumen formation, but in conjunction with moesin rather than ezrin (Georgescu et al., 2014).

In addition to extending the scaffolding capacity of ERM proteins, NHERF proteins have also been reported to regulate the activity of ERM proteins. In NHERF1/EBP50 knockout mice, levels of ERM proteins in membrane fractions of kidney and intestinal epithelial cells are decreased, suggesting that NHERF1/EBP50 stabilizes ERM proteins at the plasma membrane (Morales et al., 2004). In *Drosophila* follicle cells, the single NHERF1/2 ortholog Sip1 is thought to promote phosphorylation and activation of Moesin through recruitment of the Ste20-family kinase Slik (Hughes et al., 2010). In an ovarian cancer cell line, depletion of NHERF1/EBP50 led to reduced levels of phosphorylated ERM (pERM) upon stimulation with lysophosphatidic acid (LPA) (Oh et al., 2017). Similarly, NHERF2 was found to promote the phosphorylation of ERM in bovine pulmonary artery endothelial cells, possibly through an interaction with Rho kinase 2 (ROCK2) (Boratkó and Csortos, 2013). Finally, NHERF1/EBP50 may also indirectly affect the localization of ERM proteins, by promoting the local accumulation of PIP_2_ through recruitment of lipid phosphatases or kinases (Ikenouchi et al., 2013; Georgescu et al., 2014). Thus, NHERF proteins may function both as ERM effectors and regulators.

**FIGURE 1:**
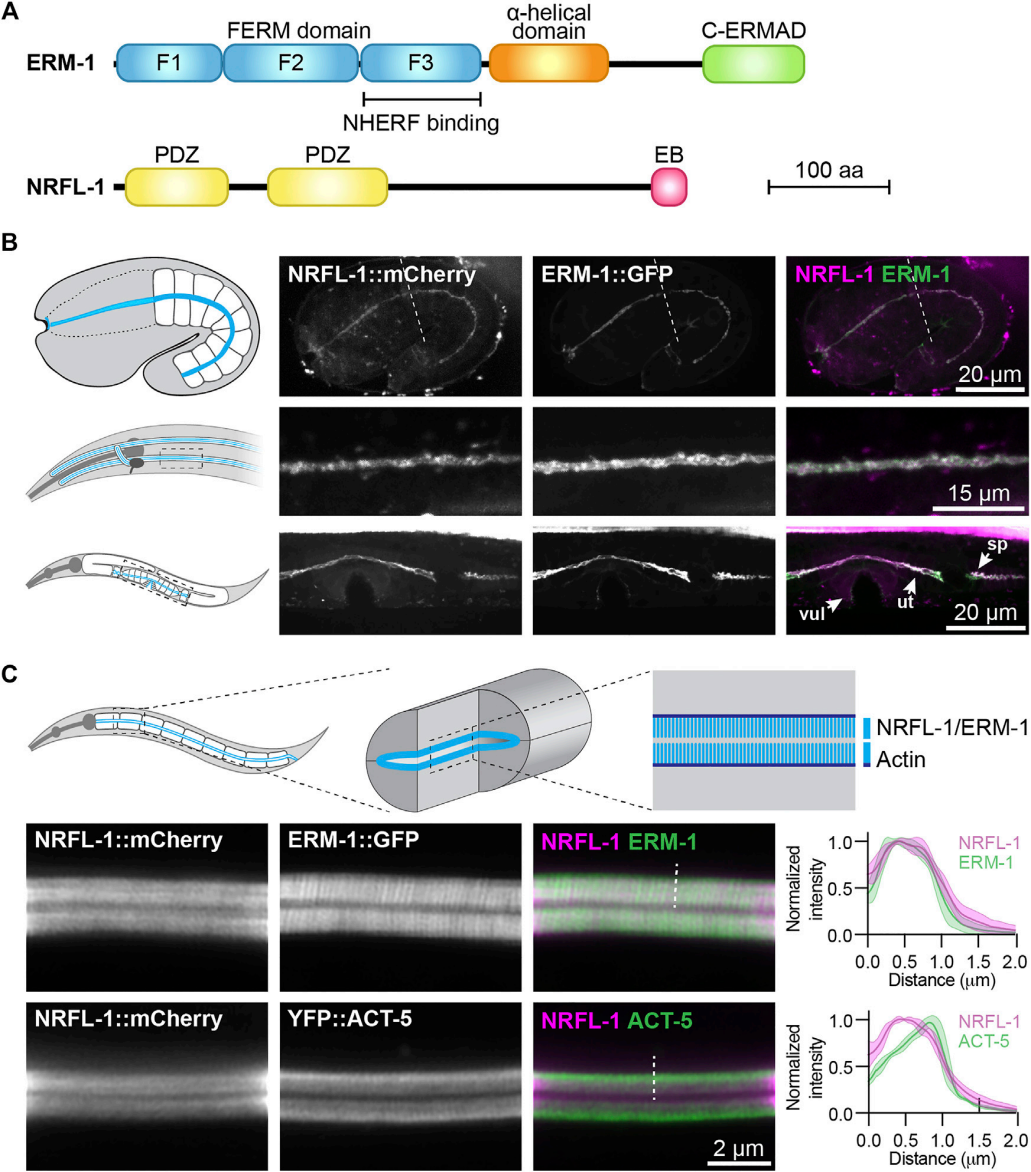
NRFL-1::mCherry localizes to the apical microvilli of intestinal cells. **(A)** Schematic representation of the domain organization of ERM-1 and NRFL-1. F1-F3 correspond to the three structural modules making up the FERM domain. FERM = Four-point-one, ezrin, radixin, moesin; C-ERMAD = C-terminal ezrin Radixin moesin (ERM) association domain; PDZ = Post-synaptic density-95, disks-large and zonula occludens-1; EB = ERM binding. **(B)** Distribution of NRFL-1::mCherry and ERM-1::GFP in embryos (top panels), the excretory canal in L1 larvae (middle panels), and the vulva (vul), uterus (ut) and spermatheca (sp) in L4 larvae (bottom panels). Dashed line in the embryo panels separates the pharynx (left) from the intestine (right). **(C)** Distribution of NRFL-1::mCherry relative to ERM-1::GFP and YFP::ACT-5 at the apical membrane of L4 larval intestines. Dashed line serves as an example of the line scan position used for the graphs on the right. Graphs plot the relative fluorescence intensity from the intestinal lumen to the cytoplasm. Solid line represents the mean and the shading lines the ± SD. *n* = 6 animals for both graphs. Images were taken using spinning-disk. **(B)** and Airyscan confocal microscopes **(C)**, and maximum intensity projections **(B)** or a single plane **(C)** are presented. Note that due to the longer wavelength emitted by mCherry compared to GFP, the microvilli are better resolved using ERM-1::GFP than using NRFL-1::mCherry.

Here, we make use of the nematode *Caenorhabditis elegans* to better understand how NHERF and ERM proteins function together to promote apical domain identity. The *C. elegans* genome encodes single orthologs of each protein family, termed NRFL-1 and ERM-1, that are highly similar in sequence and domain composition to their counterparts in other organisms (Figure 1A; Supplementary Figure S1A). ERM-1 localizes to the apical surface of several epithelial tissues and is essential for apical membrane morphogenesis in the intestine (Göbel et al., 2004; Van Fürden et al., 2004). Loss of *erm-1* in the intestine causes constrictions, loss of microvilli, severe reduction in the levels of apical actin, and defects in the accumulation of junctional proteins (Göbel et al., 2004; Van Fürden et al., 2004; Bernadskaya et al., 2011). Recently, we demonstrated that the functioning of ERM-1 critically depends on its ability to bind membrane phospholipids, while phosphorylation of a C-terminal regulatory threonine residue modulates ERM-1 apical localization and dynamics (Ramalho et al., 2020).

In contrast to ERM-1, little is known about the functioning of NRFL-1. A yeast-two hybrid screen identified the amino acid transporter (AAT) family protein AAT-6 as an interactor of NRFL-1 (Hagiwara et al., 2012). However, the effects of NRFL-1 loss are minor. In aging adults, AAT-6 is no longer retained at the luminal membrane of the intestine in *nrfl-1* mutants, while younger *nrfl-1* mutants show increased mobility of AAT-6 by fluorescence recovery after photobleaching (FRAP). Moreover, *nrfl-1* mutants are homozygous viable, demonstrating that NRFL-1 is not critical for intestinal development (Hagiwara et al., 2012).

To investigate the relationship between ERM-1 and NRFL-1, we used CRISPR/Cas9 engineering to generate an *nrfl-1* deletion mutant, a mutant lacking the ERM-1 binding domain, and fluorescently tagged NRFL-1 variants. We show that NRFL-1 localizes to the apical microvillar domain of the intestine, and that this localization depends on the ability of NRFL-1 to bind to ERM-1 via the C-terminal ERM-1 binding domain. The loss of *nrfl-1* did not affect the localization, phosphorylation status, or protein dynamics of ERM-1, indicating that *C. elegans* NRFL-1 does not control the activity of ERM-1. However, when we combined the *nrfl-1* null mutant with *erm-1* mutants that block or mimic phosphorylation of the C-terminal threonine 544 residue, we observed severe intestinal defects, resembling the effects of strong loss of *erm-1* function. In mice, ezrin was shown to form distinct complexes with NHERF1/EBP50 and actin. As the ERM-1 phosphorylation mutants affect the ability of ERM-1 to interact with actin, we postulate that the activities of ERM-1 in the intestine redundantly involve actin binding and the organization of apical domain composition through NRFL-1.

## Results

### NRFL-1 Localizes to the Apical Domain Through ERM-1 Binding

To investigate the relationship between NRFL-1 and ERM-1, we first examined if NRFL-1 colocalizes with ERM-1. We used CRISPR/Cas9 to engineer an endogenous C-terminal NRFL-1::mCherry fusion, which tags all predicted isoforms. Animals homozygous for the *nrfl-1::mCherry* knock-in are viable and have a wild-type appearance. We detected expression of NRFL-1 in multiple epithelia including the intestine, excretory canal, pharynx, uterus, and spermatheca (Figures 1B,C). In each of these tissues, NRFL-1::mCherry co-localized with an endogenous ERM-1::GFP fusion protein at the cortex (Figures 1B,C). In the embryo, NRFL-1 localized to the nascent apical domain of intestinal cells, overlapping with ERM-1 (Figure 1B). Confocal super resolution imaging of the intestine in larval stages showed co-localization of NRFL-1 with ERM-1::GFP and YFP::ACT-5 at microvilli, apical to the more intense belt of YFP::ACT-5 at the terminal web (Figure 1C). The observed distribution of NRFL-1::mCherry is consistent with previous observations in *C. elegans* (Hagiwara et al., 2012), as well as with localization of EBP50 in mammalian epithelial tissues (Ingraffea, 2002; Morales et al., 2004; Kreimann et al., 2007).

**FIGURE 2:**
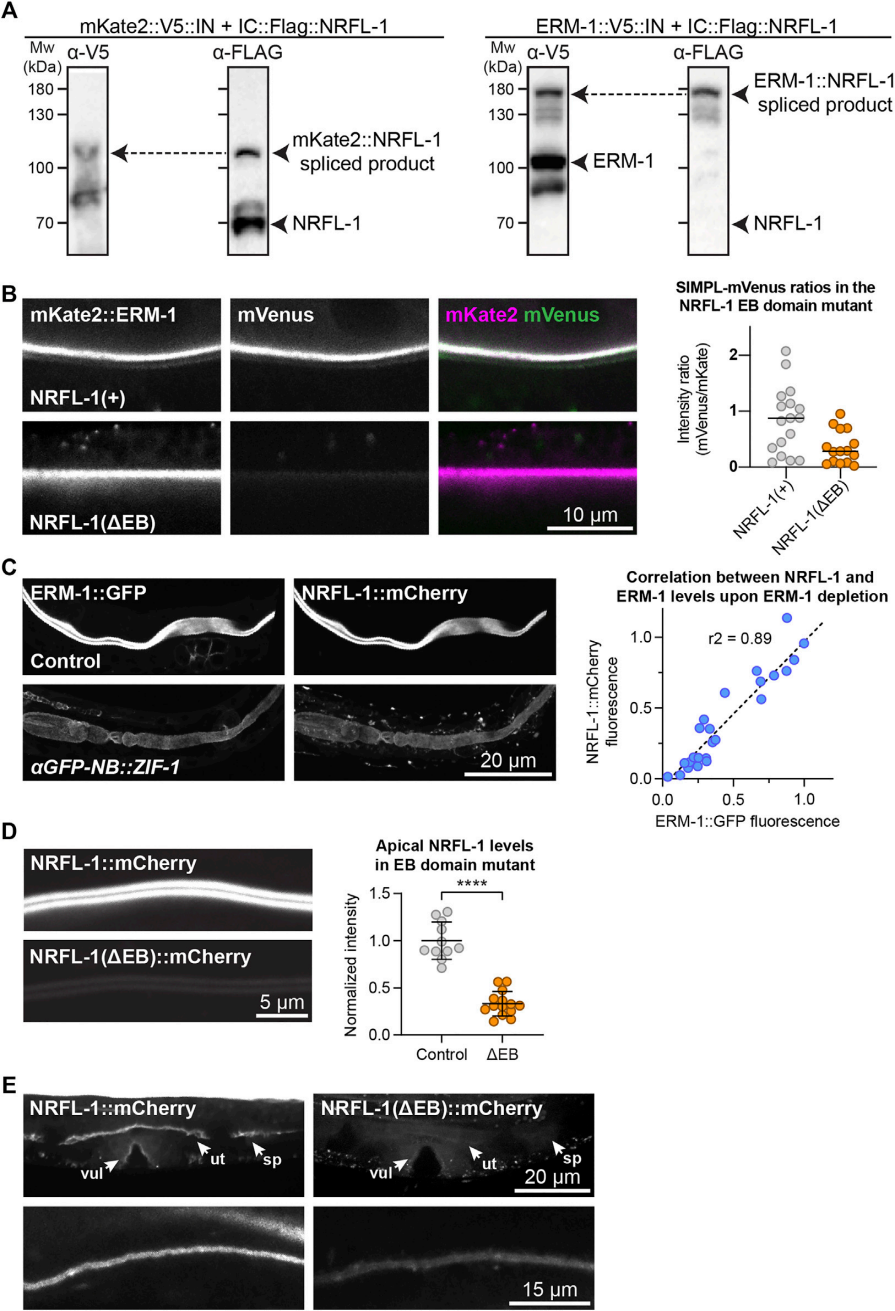
NRFL-1 localizes to the apical domain through ERM-1 binding. **(A)** Detection of an ERM-1–NRFL-1 interaction using the SIMPL system. V5 and FLAG epitopes are detected by western blot. Arrowheads indicate both unspliced proteins and the higher molecular weight covalently linked fusion proteins, generated by Intein splicing activity. Little splicing of NRFL-1 is observed with the control mKate2::V5::IN protein, while all NRFL-1 is spliced to ERM-1 in animals expressing ERM-1::V5::IN **(B)** Detection of an interaction of ERM-1 with wild-type NRFL-1, but not with NRFL-1(ΔEB), using the SIMPL-mVenus system. NRFL-1a::InteinC-3xFLAG-VC155 [NRFL-1(+)] or NRFL-1a(ΔEB)::InteinC-3xFLAG-VC155 [NRFL-1(ΔEB)] are expressed with mKate2::ERM-1::VN155-HA-V5-InteinN (mKate2::ERM-1). Fluorescence micrographs show representative examples. Graphs show quantification of apical mVenus levels, expressed as a ratio over mKate2::ERM-1 to account for varying expression levels of the extrachromosomal array. Each data point represents a single intestinal cell. Lines indicate median. N = 17 cells for NRFL-1(+) and 15 cells for NRFL-1(ΔEB). **(C)** Quantification of apical levels of NRFL-1::mCherry *vs*. ERM-1::GFP in L1 larval intestines upon different levels of ERM-1::GFP depletion by expression of an anti-GFP nanobody::ZIF-1 fusion protein. Fluorescence micrographs show representative examples, graph shows quantification of signal intensity at the apical membrane. Each data point in the graph represents a single animal, and the line a linear regression. Values are normalized to the mean intensity in control animals. *n* = 25 animals. **(D)** Quantification of apical levels of NRFL-1(ΔEB)::mCherry relative to NRFL-1::mCherry at the apical membrane of L1 larval intestines. Fluorescence micrographs show representative examples, and the graph the quantification. Each data point in the graph represents a single animal, and values are normalized to the mean intensity in control animals. Error bars: mean ± SD; Statistical test: Welch’s Student’s t-test; **** = *p* ≤ 0.0001. *n* = 10 animals for NRFL-1::mCherry and 14 animals for NRFL-1(ΔEB)::mCherry. **(E)** Localization of NRFL-1::mCherry and NRFL-1(ΔEB)::mCherry in the vulva (vul), uterus (ut) and spermatheca (sp) in L4 larvae (top panels), and the excretory canal in L1 larvae (bottom panels). Images of the same tissue were acquired and displayed with the same settings for comparison. All images were taken using a spinning disk confocal microscope, and a single plane **(B)** or maximum intensity projections **(C,D, and E)** are presented.

We previously showed that ERM-1 and NRFL-1 interact in a yeast two-hybrid assay and in pull-downs from mammalian cultured cells (Koorman et al., 2016). To determine if these proteins interact in a more physiological setting, we used the recently developed split intein-mediated protein ligation (SIMPL) system that relies on protein splicing by split intein domains to detect protein–protein interactions (Yao et al., 2020). We ubiquitously expressed ERM-1 fused to the intein N-terminal fragment (IN) and the V5 epitope, and NRFL-1 fused to the C-terminal fragment (IC) and the FLAG epitope. We observed full splicing of NRFL-1 to ERM-1 by western blot of *C. elegans* lysates, apparent as a high molecular weight band that stains with both V5 and FLAG antibodies (Figure 2A). In contrast, a negative control pair consisting of IC-tagged NRFL-1 and IN-tagged mKate2 showed only limited splicing of NRFL-1 to mKate2 (Figure 2A). To visualize if splicing occurs *in vivo* in the intestine, we modified the SIMPL system by including a split mVenus tag. We added the mVenus N-terminal fragment (VN155) to ERM-1::V5-IN and mVenus C-terminal fragment (VC155) to IC-FLAG::NRFL-1, such that upon intein splicing the reconstituted mVenus becomes linked to NRFL-1 (Kodama and Hu, 2010). We readily observed localization of mVenus at the apical domain of intestinal cells, indicating that NRFL-1 and ERM-1 interact in this tissue (Figure 2B).

We next investigated whether NRFL-1 distribution to the apical plasma membrane is dependent on ERM-1, by analyzing NRFL-1::mCherry upon tissue-specific depletion of ERM-1. To deplete ERM-1 in intestinal cells, we introduced an anti-GFP-nanobody::ZIF-1 fusion driven by the intestine-specific *elt-2* promoter as an extrachromosomal array in animals expressing endogenous ERM-1::GFP and NRFL-1::mCherry (Wang et al., 2017). Expression of the nanobody::ZIF-1 fusion resulted in variable levels of ERM-1::GFP depletion. The apical levels of ERM-1::GFP and NRFL-1::mCherry showed a linear correlation, indicating that apical recruitment of NRFL-1 in the intestine directly depends on ERM-1 (Figure 2C).

The interaction between mammalian EBP50 and ezrin requires the C-terminal EB domain (Reczek et al., 1997; Reczek and Bretscher, 1998; Finnerty et al., 2004), which is conserved in NRFL-1 (Figures 1A; Supplementary Figure S1A). To determine if the NRFL-1 EB domain is required for the interaction with ERM-1, we repeated the SIMPL-mVenus experiment using an NRFL-1(ΔEB) mutant that lacks the C-terminal 28 amino acids of NRFL-1. Compared to wild-type NRFL-1, we observed only residual apical localization of mVenus in intestinal cells, indicating that the interaction of NRFL-1 with ERM-1 depends on the presence of the EB domain (Figure 2B).

To determine if the EB domain is necessary for the apical localization of NRFL-1, we used CRISPR/Cas9 to engineer the 28 aa EB deletion in the *nrfl-1::mCherry* strain. The resulting *nrfl-1(Δeb)::mCherry* animals are homozygous viable, consistent with the lack of severe defects in previously described *nrfl-1* mutants (Hagiwara et al., 2012; Na et al., 2017). We detected a dramatic reduction in apical levels of NRFL-1(ΔEB)::mCherry in intestinal cells when compared with NRFL-1::mCherry (Figure 2D). NRFL-1(ΔEB)::mCherry also failed to localize at the cortex in the uterus and spermatheca, while apical levels in the excretory canal were reduced (Figure 2E). These results indicate that apical recruitment of NRFL-1 is mediated by the EB domain. However, the presence of some residual apical NRFL-1(ΔEB)::mCherry in the intestine and excretory canal suggests the existence of alternative membrane-targeting mechanisms. Collectively, our results show that the interaction between ERM and NHERF proteins is conserved in *C. elegans*, and that the localization of NRFL-1 is largely mediated by its interaction with ERM-1.

### NRFL-1 Cooperates with ERM-1 Phosphorylation in Regulating Intestinal Lumen Formation

**FIGURE 3:**
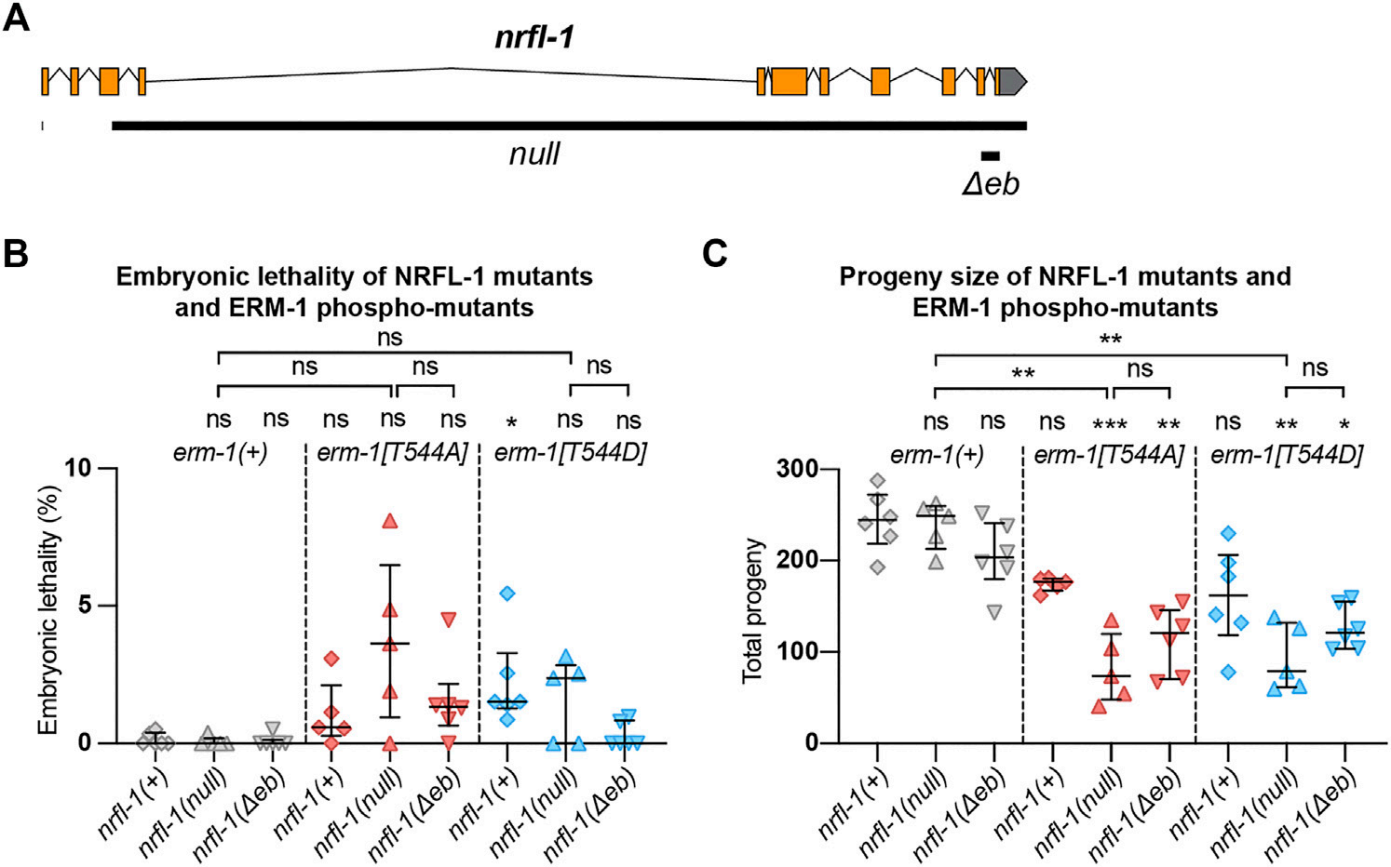
NRFL-1 cooperates with ERM-1 C-terminal phosphorylation. **(A)** Gene model for *nrfl-1a*. Orange boxes represent exons and lines represent introns. Grey box represents 3′ untranslated region. Black bars denote the regions deleted in *null* and *Δeb* alleles. **(B,C)** Quantification of embryonic lethality **(B)** and total progeny **(C)** from parents of indicated genotypes. Each data point represents the embryonic lethality **(B)** or progeny **(C)** of a single animal; N = 5 or 6. Error bars: mean ± SD. Statistical test: Kruskal-Wallis test with Dunn’s multiple comparison correction.

We next wanted to investigate the effects of loss of NRFL-1 on intestinal lumen formation. Previous studies using partial deletion alleles of *nrfl-1* indicated that loss of NRFL-1 alone does not cause defects in the formation of the intestine (Hagiwara et al., 2012; Na et al., 2017). To rule out the possibility that the lack of severe defects is due to the production of truncated NRFL-1 proteins, we used CRISPR/Cas9 genome engineering to generate the *nrfl-1(mib59)* deletion allele. This allele lacks almost the entire *nrfl-1* locus and additionally causes a frameshift in the first exon of the long isoforms (Figure 3A). Hence, we refer to *mib59* as *nrfl-1(null)*. The *mib59* deletion also removes a candidate non-coding RNA and overlapping 21U-RNA located in the large 3^rd^ exon of *nrfl-1a.* Animals homozygous for the *nrfl-1(null)* allele are viable, have a healthy appearance, and normal brood sizes, confirming that NRFL-1 is not essential for *C. elegans* development (Figure 3B,C).

One of the possible reasons for the lack of a severe intestinal phenotype in *nrfl-1(null)* animals is that NRFL-1 may only mediate part of the functions of ERM-1 in the intestine. To investigate this possibility, we made use of the non-phosphorylatable *erm-1[T544A]* and phosphomimetic *erm-1[T544D]* alleles we generated previously (Ramalho et al., 2020). Both mutants cause a delay in the apical recruitment of ERM-1 and actin during embryogenesis, and the appearance of constrictions along the course of the lumen that only occasionally persist to the L1 stage. In contrast to *erm-1* RNAi or strong loss-of-function alleles, however, these animals are viable. Thus, *erm-1[T544A]* and *erm-1[T544D]* represent partial loss-of-function alleles that may act as a sensitized background to reveal the contribution of NRFL-1 to ERM-1 functioning. We therefore generated double mutants that carry the *nrfl-1(null)* allele and either of the *erm-1[T544A]* or *erm-1[T544D]* alleles. As a first indicator of synthetic defects, we examined the double mutant strains for embryonic lethality or an increase in the mild brood size defect observed in *erm-1[T544A]* and *erm-1[T544D]* mutants. We did not observe strong embryonic lethality in any mutant combination (<5%, Figure 3B). However, combining *nrfl-1(null)* with either *erm-1* phosphorylation mutant resulted in a strongly reduced brood size (Figure 3C). In addition, many larvae in the double mutant combination had a sick appearance and developed slowly. Nevertheless, both double mutants can be maintained as homozygotes, unlike strong *erm-1* loss of function mutants.

**FIGURE 4:**
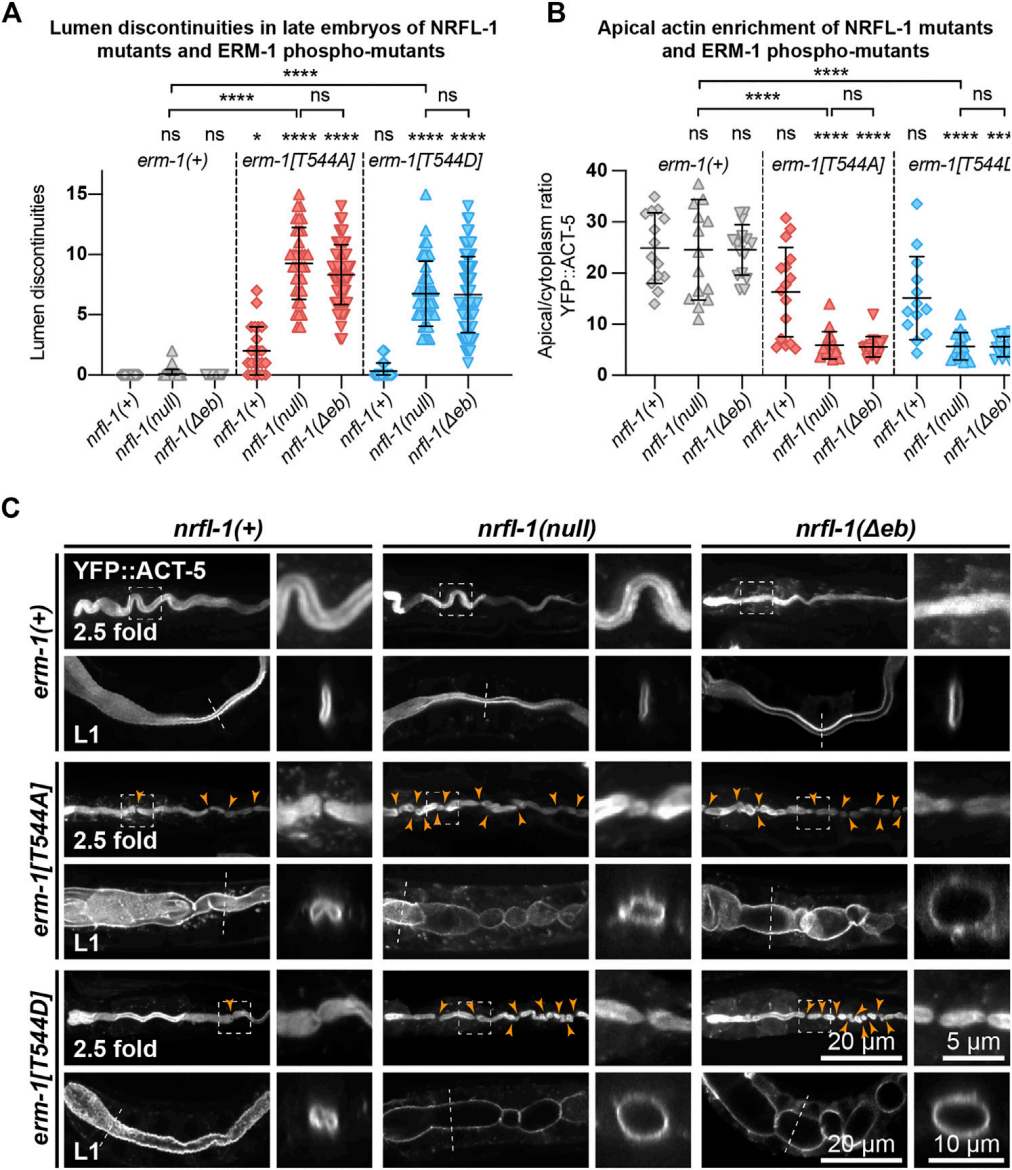
ERM-1 phosphorylation and NRFL-1 redundantly contribute to intestinal morphology. **(A)** Quantification of lumen discontinuities in 2.5-fold stage embryos of indicated genotypes expressing YFP::ACT-5. Each data point represents a single animal. Error bars: mean ± SD. Statistical test: Kruskal-Wallis test with Dunn’s multiple comparison correction. *nrfl-1(+); erm-1(+) n* = 19, *nrfl-1(null); erm-1(+) n* = 35, *nrfl-1(Δeb); erm-1(+) n* = 48, *nrfl-1(+); erm-1[T544A] n* = 23, *nrfl-1(null); erm-1[T544A] n* = 35, *nrfl-1(Δeb); erm-1[T544A] n* = 61, *nrfl-1(+); erm-1[T544D] n* = 28, *nrfl-1(null); erm-1[T544D] n* = 41, *nrfl-1(Δeb); erm-1[T544D] n* = 69. **(B)** Quantification of the apical–cytoplasm ratio of YFP::ACT-5 in L1 larvae of indicated genotypes. Each data point represents a single animal. Error bars: mean ± SD. Statistical test: Kruskal-Wallis test with Dunn’s multiple comparison correction. *nrfl-1(+); erm-1(+) n* = 14, *nrfl-1(null); erm-1(+) n* = 15, *nrfl-1(Δeb); erm-1(+) n* = 16, *nrfl-1(+); erm-1[T544A] n* = 16, *nrfl-1(null); erm-1[T544A] n* = 16, *nrfl-1(Δeb); erm-1[T544A] n* = 16, *nrfl-1(+); erm-1[T544D] n* = 13, *nrfl-1(null); erm-1[T544D] n* = 16, *nrfl-1(Δeb); erm-1[T544D] n* = 16. **(C)** Representative images of intestinal defects in 2.5-fold stage embryos and L1 larvae of indicated genotypes, expressing YFP::ACT-5 as an apical marker. Images of the 2.5-fold stage embryos were computationally straightened, and the orange arrowheads indicate the constrictions in the lumen. Small panels to the right of each embryo panel show an enlargement of the region indicated by the dashed box, and small panels to the right of each L1 larva show a cross-section view of the intestine at the position indicated by the dotted line. All images are taken using a spinning-disk confocal microscope, and maximum intensity projections are presented.

We next examined the formation of the intestinal lumen and actin distribution using YFP::ACT-5 as a marker. We did not detect any defects in apical enrichment of ACT-5 or intestinal morphology in *nrfl-1(null)* embryos and larvae (Figures 4A–C). Combining the *erm-1[T544A]* and *erm-1[T544D]* alleles with the *nrfl-1(null)* allele significantly increased the frequency of intestinal constrictions and their persistence until larval development (Figures 4A,C). Intestines of early larval *nrfl-1(null); erm-1[T544A]* and *nrfl-1(null); erm-1[T544D]* animals were characterized by a cystic appearance and multiple constrictions that block intestinal flow as seen in feeding assays with fluorescent membrane-impermeable dextran (Supplementary Figure S2A). In surviving L2 or older animals, we only observed morphological defects but no lumen discontinuities, indicating that the early larval arrest in double mutants is due to a block of flow of food through the intestine (Supplementary Figure S2B). In addition to the increase in intestinal constrictions, we also observe that loss of *nrfl-1* caused a further decrease in the apical levels of YFP::ACT-5 in *erm-1[T544A]* and *erm-1[T544D]* mutant animals (Figure 4B).

Finally, as the EB domain is essential for the apical localization of NRFL-1 and its interaction with ERM-1, we determined if loss of the EB domain results in similar synergistic phenotypes with the ERM-1 phosphorylation mutants as complete loss of NRFL-1. We used CRISPR/Cas9 genome engineering to generate a second *nrfl-1(Δeb)* allele, also removing the final 28 aa but lacking the mCherry tag used above (Figure 3A). Similar to our observations for the mCherry-tagged variant, homozygous *nrfl-1(Δeb)* mutants are viable and show no significant defects in brood size, intestinal development, or apical ACT-5 enrichment (Figures 3B,C; Figures 4A–C). However, when combined with *erm-1[T544A]* or *erm-1[T544D]*, the resulting double mutants showed similar defects in viability, growth, brood size, intestinal development, and ACT-5 enrichment as observed using the *nrfl-1(null)* allele (Figures 3B,C; Figures 4A–C). Thus, the *nrfl-1(Δeb)* allele behaves like a null allele of *nrfl-1*. Taken together, our data show that NRFL-1 and ERM-1 function together in promoting lumen formation in the *C. elegans* intestine, and the binding to ERM-1 is essential for the functioning of NRFL-1 in the intestine.

### NRFL-1 Does Not Directly Regulate ERM-1 Activity

**FIGURE 5:**
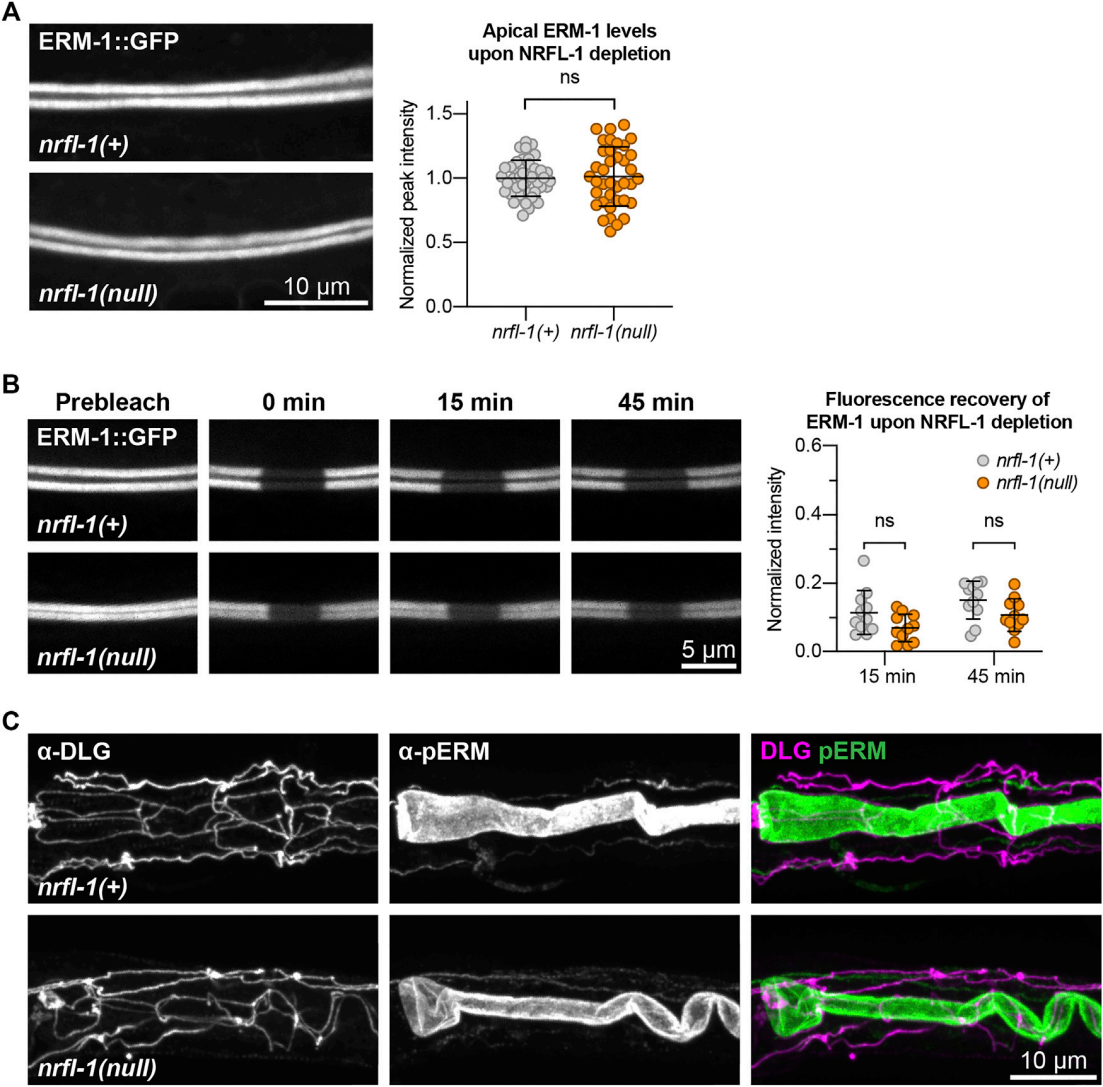
NRFL-1 does not regulate ERM-1 apical accumulation, dynamics, or phosphorylation status. **(A)** Representative images and quantification of ERM-1::GFP levels at the apical membrane of intestines in *nrfl-1(+)* and *nrfl-1(null)* L4 larvae. Each data point represents a single animal, and values are normalized to the mean intensity in control animals. Error bars: mean ± SD. Statistical test: Unpaired Student’s t-test. *nrfl-1(+) n* = 40, *nrfl-1(null) n* = 38. **(B)** FRAP analysis of apical ERM-1::GFP in the intestine of *nrfl-1(+)* and *nrfl-1(null)* L4 larvae. Fluorescence micrographs show representative examples. Graph shows the fluorescence intensity of ERM-1 in the photobleached region at the apical intestinal domain during recovery. Each data point represents a single animal, and values are relative to prebleach levels. Error bars: mean ± SD. Statistical test: Unpaired Student’s t-test. *n* = 11 for both genotypes and both timepoints. **(C)** Representative images of fixed *nrfl-1(+)* and *nrfl-1(null)* larvae stained with antibodies recognizing the junctional protein DLG-1 (α-DLG) and phosphorylated ERM-1 (α-pERM).

NRFL-1 could function together with ERM-1 in at least two ways. It could act as a scaffold protein that is required for ERM-1 to organize protein complexes at the membrane, or it could regulate the activity of ERM-1 itself. To distinguish between these possibilities, we investigated whether loss of NRFL-1 affects the distribution, mobility, or T544 phosphorylation status of ERM-1. We first analyzed the distribution of ERM-1::GFP in larval *nrfl-1(null)* mutants. We did not detect any change in ERM-1::GFP subcellular localization or levels at the apical membrane in the intestine (Figure 5A). Moreover, FRAP analysis demonstrated that the mobility of ERM-1::GFP at the apical intestinal membrane was not significantly altered in *nrfl-1(null)* larvae (Figure 5B). We next investigated whether NRFL-1 regulates ERM-1 C-terminal phosphorylation by staining *nrfl-1(null)* mutants with an antibody specific for the C-terminal phosphorylated form of ERM proteins (pERM). The residues used to raise this antibody are fully conserved between mammals and *C. elegans* (Ramalho et al., 2020). Nevertheless, we first confirmed the specificity of the antibody for T544 phosphorylated ERM-1 by immunostaining of ERM-1[T544A] mutant animals. We readily detected pERM staining of the intestinal lumen in wild-type larvae, while no staining was observed in ERM-1[T544A] animals (Supplementary Figure S3A). Moreover, treatment of embryos with a phosphatase abolished staining with the pERM antibody (Supplementary Figure S3B). Thus, the pERM antibody is specific for T544 phosphorylated ERM-1. We then stained *nrfl-1(+)* and *nrfl-1(null)* animals with the pERM antibody. In both backgrounds, the pERM antibody stained the lumen of the intestine, indicating that loss of *nrfl-1* does not significantly alter the phosphorylation status of the C-terminal regulatory threonine of ERM-1 (Figure 5C). Taken together, our results show that NRFL-1 does not regulate the distribution, dynamics, or phosphorylation of ERM-1, and therefore does not seem to directly regulate ERM-1.

## Discussion

ERM and NHERF proteins function together in the specialization of polar membrane domains in several mammalian cell types. Here, we show that this cooperation is conserved in *C. elegans*, and that ERM-1 and NRFL-1 function together in lumen formation in the intestine. NRFL-1 physically interacts with ERM-1 through its C-terminal EB domain. The interaction with ERM-1 is responsible for the apical localization of NRFL-1 in the intestine, as depletion of ERM-1 or deletion of the EB domain results in a loss of NRFL-1 apical localization.

Loss of *nrfl-1* by itself did not cause overt defects in intestinal formation, animal development, or viability. Three previous partial deletion alleles of *nrfl-1* have been described: *ok2292, tm3501,* and *ok297* (Hagiwara et al., 2012, 1; Na et al., 2017). No severe defects in animal development were reported for *ok2292* or *tm3501* (Hagiwara et al., 2012). However, *nrfl-1(ok297)* animals were reported to have ruptured vulva and sterile phenotypes (Na et al., 2017). Given that neither of the other two previously characterized alleles nor our newly generated *nrfl-1* deletion allele display these phenotypes, we think it is likely that the *ok297* strain analyzed either contains additional background mutations or that *ok297* represents a neomorphic allele of *nrfl-1*. The non-essential role of *nrfl-1* contrasts with data in mice, where NHERF1/EBP50 loss causes defects in intestinal microvilli formation (Morales et al., 2004), and in *Drosophila,* where *Sip1* mutants cause morphological defects in the follicle cells surrounding the oocytes and late embryonic lethality (Hughes et al., 2010).

Combining the *nrfl-1(null)* deletion mutant with phosphorylation-defective *erm-1[T544A]* or *erm-1[T544D]* mutants resulted in severe defects in intestinal lumen formation. Double mutant animals have a cystic intestinal lumen, characterized by distended regions and severe constrictions. These animals develop slowly or arrest during early larval development, likely due at least in part to the inability of luminal contents to travel through the digestive system. The double mutant intestinal phenotype is similar to that described for *erm-1(RNAi)* and the *erm-1(tm677)* deletion allele (Göbel et al., 2004; Van Fürden et al., 2004), and to an ERM-1 mutant unable to bind to the plasma membrane (ERM-1[4 KN]) (Ramalho et al., 2020). Nevertheless, complete loss of *erm-1* functioning causes maternal effect L1 lethality, while *erm-1[T544A]; nrfl-1* and *erm-1[T544D]; nrfl-1* double mutant strains can be maintained homozygously despite the developmental defects.

In many other systems, the loss of NHERF proteins results in similar phenotypes as loss of ERM proteins. NHERF1/EBP50 and ezrin are both required for microvilli formation in mouse intestinal cells as well as in cultured epithelial cells (Bonilha et al., 1999; Morales et al., 2004; Saotome et al., 2004; Garbett et al., 2010; LaLonde et al., 2010; Viswanatha et al., 2012), and loss of NHERF1/EBP50 or moesin causes similar defects in the morphogenesis of 3D cysts grown from Caco-2 cells (Georgescu et al., 2014). This is likely due to positive effects of NHERF proteins on the localization, stability, or activity of ERM proteins (Morales et al., 2004; Hughes et al., 2010; Boratkó and Csortos, 2013; Oh et al., 2017). In *C. elegans* we found no evidence for such a role towards wild-type ERM-1. The loss of NRFL-1 did not cause any noticeable defects in the localization or levels of ERM-1 at the apical membrane, in the mobility of ERM-1 as examined by FRAP, or in the phosphorylation of T544. We also did not observe a decrease in apical actin levels in *nrfl-1* mutant animals. In organisms where NHERF loss affects the localization or activity of ERM proteins, the loss of NHERF would result in both a lack of protein scaffolding by NHERF and a reduction in actin organizing ability of ERM. Thus, the lack of a reciprocal relationship in *C. elegans* make it a unique model in which these different aspects of ERM protein function can be observed separately.

To explain our observations, we considered two possible models for the roles of ERM-1 and NRFL-1. In the first, the functioning of *C. elegans* ERM-1 involves at least two separable activities: one regulated by the phosphorylation of the C-terminal T544 residue, and one mediated via the recruitment of NRFL-1. The exact consequences of altering T544 phosphorylation are not known, but apical enrichment of the intestinal actin ACT-5 is clearly disrupted (Ramalho et al., 2020). This is in agreement with findings in other systems that C-terminal phosphorylation of ERM proteins is required for apical recruitment of actin (Hipfner et al., 2004; Roch et al., 2010; Abbattiscianni et al., 2016). Interestingly, fractionation experiments from kidney epithelial cells indicated that ERM proteins interact with actin and NHERF1/EBP50 in distinct complexes (Morales et al., 2004). Together with the lack of ACT-5 defects in *nrfl-1* mutants, this presents a possible model in which T544 phosphorylation regulates actin binding, while the scaffolding activities of NRFL-1 mediate recruitment or local distribution of membrane-associated proteins by ERM-1. This model can, however, not account for the observation that loss of *nrfl-1* causes a further decrease of apical actin levels in *erm-1[T544A]* or *erm-1[T544D]* mutant animals, which indicates that NRFL-1 can contribute to the actin organizing activities of ERM-1.

In the second model, both the T544 phosphorylation cycle and binding of NRFL-1 promote an open, active, ERM-1 configuration. A redundant role for T544 phosphorylation and NRFL-1 binding in ERM-1 activation would account for the lack of effects of *nrfl-1* loss on wild-type ERM-1, and for our previous observations that T544 mutations in *C. elegans* have a relatively mild effect on ERM-1 activity compared to similar mutations in mammalian ERM proteins (Ramalho et al., 2020). Support for this model comes from the wedge mechanism that has been proposed for the mammalian kinase LOK, in which the C-terminal domain of LOK wedges apart the FERM and F-actin-binding domains of ezrin to gain access to the regulatory T567 site (Pelaseyed et al., 2017). A chimeric kinase in which the LOK C-terminal domain was replaced with the NRFL-1 ortholog EBP50t was able to phosphorylate ezrin, indicating that EBP50t harbors a similar wedging activity as the LOK C-terminal domain. However, the existence of a wedging mechanism has not been investigated in *C. elegans* nor independently confirmed in mammalian systems. Moreover, loss of *nrfl-1* alone did not affect apical actin levels. Together with the lack of effects of *nrfl-1* loss on ERM-1 localization and stability, this argues against this second model: if T544 phosphorylation and NRFL-1 performed similar roles in ERM-1 activation, their loss would be expected to result in similar defects as well.

Most likely, the activities of NRFL-1 and ERM-1 in *C. elegans* involve a combination of these two models. NRFL-1 may primarily mediate the scaffolding activities of ERM-1 but also promote the open and active conformation of ERM-1, while T544 phosphorylation is the dominant mechanism regulating actin organization by ERM-1. Only when T544 phosphorylation is disrupted does the positive effect of NRFL-1 binding on promoting an open ERM-1 conformation capable of actin binding become apparent. Regardless of the exact mechanism, our results demonstrate that ERM-1 phosphorylation and NRFL-1 redundantly control lumen formation in the *C. elegans* intestine.

There are important differences between our studies in *C. elegans* and studies of ERM proteins in other organisms. The first is that phosphorylation of the C-terminal threonine residue is generally considered to be a critical step in the activation of ERM proteins, while T544A and T544D mutations are tolerated in *C. elegans.* Importantly, the requirement for phosphorylation is not universal. Several studies have observed rescuing activity of Moesin-T559A or Moesin-T559D transgenes in *Drosophila* (Speck et al., 2003; Hipfner et al., 2004; Roch et al., 2010), and phosphorylation of ERM proteins is not required for the formation of microvilli-like structures in A431 and MDCK II cells (Yonemura et al., 2002). The second major difference is that loss of *nrfl-1* by itself causes no severe defects in *C. elegans*, while loss of NHERF1/EBP50 causes intestinal abnormalities in mice (Morales et al., 2004; Broere et al., 2009) and flies lacking the NHERF ortholog Sip1 are not viable (Hughes et al., 2010). We think it is most likely that the activities and regulation of ERM proteins are conserved between organisms—involving lipid binding, regulatory phosphorylation on the C-terminal threonine residue, and the binding to adapter proteins—but that the relative importance of these events depends on the biological setting and experimental system used.

## Materials and Methods

### *C. elegans* Strains and Culture Conditions

*C. elegans* strains were cultured under standard conditions (Brenner, 1974). Only hermaphrodites were used, and all experiments were performed with animals grown at 15 °C or 20 °C on standard Nematode Growth Medium (NGM) agar plates seeded with OP50 *Escherichia coli*. Table 1 contains a list of all the strains used.

**TABLE 1:** List of *C. elegans* strains used

Strain	Genotype
N2	*Wild type*
JM125	*caIs107[Pges-1::YFP::act-5]*
BOX163	*erm-1(mib9[erm-1[p. T544D]) I*
BOX165	*erm-1(mib10[erm-1[p. T544A]) I*
BOX196	*erm-1(mib10[erm-1[p. T544A]) I; caIs107[Pges-1::YFP::act-5]*
BOX197	*erm-1(mib9[erm-1[p.T544D]) I; caIs107[Pges-1::YFP::act-5]*
BOX213	*erm-1(mib15[erm-1::GFP]) I*
BOX273	*mibIs48[Pelt-2::TIR-1::tagBFP2-Lox511::tbb-2-3′UTR, IV::5014740-5014802 (cxTi10816 site)]) IV*
BOX404	*nrfl-1(mib59[nrfl-1a(null = c.-1_8del; c.287_1404+703)]) IV*
BOX422	*nrfl-1(mib73[nrfl-1::mCherry]) IV; mibIs48[Pelt-2::TIR-1::tagBFP2-Lox511::tbb-2-3′UTR, IV:5014740-5014802 (cxTi10816 site)]) IV*
BOX428	*erm-1(mib15[erm-1::GFP]) I; nrfl-1(mib73[nrfl-1::mCherry]) IV; mibIs48[Pelt-2::TIR-1::tagBFP2-Lox511::tbb-2-3′UTR, IV:5014740-5014802 (cxTi10816 site)]) IV*
BOX429	*nrfl-1(mib73[nrfl-1::mCherry]) IV; mibIs48[Pelt-2::TIR-1::tagBFP2-Lox511::tbb-2-3′UTR, IV:5014740-5014802 (cxTi10816 site)]) IV; caIs107[Pges-1::YFP::act-5]*
BOX440	*nrfl-1(mib75[nrfl-1a(Δeb = c.1318_1401del)::mCherry]) IV; mibIs48[Pelt-2::TIR-1::tagBFP2-Lox511::tbb-2-3′UTR, IV:5014740-5014802 (cxTi10816 site)]) IV; caIs107[Pges-1::YFP::act-5]*
BOX495	*erm-1(mib15[erm-1::GFP]) I; nrfl-1(mib59[nrfl-1a(null c.-1_8del; c.287_1404+703)]) IV; mibIs48[Pelt-2::TIR-1::tagBFP2-Lox511::tbb-2-3′UTR, IV:5014740-5014802 (cxTi10816 site)] IV*
BOX597	*nrfl-1(mib104[nrfl-1a(Δeb = c.1318_1401del)]) IV*
BOX670	*erm-1(mib10[erm-1[p.T544A]) I; nrfl-1(mib59[nrfl-1a(null = c.-1_8del; c.287_1404+703)]) IV*
BOX671	*erm-1(mib9[erm-1[p.T544D]) I; nrfl-1(mib59[nrfl-1a(null = c.-1_8del; c.287_1404+703)]) IV*
BOX672	*nrfl-1(mib59[nrfl-1a(null = c.-1_8del; c.287_1404+703)]) IV; caIs107[Pges-1::YFP::act-5]*
BOX673	*erm-1(mib10[erm-1[p.T544A]) I; nrfl-1(mib59[nrfl-1a(null = c.-1_8del; c.287_1404+703)]) IV; caIs107[Pges-1::YFP::act-5]*
BOX674	*erm-1(mib9[erm-1[p.T544D]) I; nrfl-1(mib59[nrfl-1a(null = c.-1_8del; c.287_1404+703)]) IV; caIs107[Pges-1::YFP::act-5]*
BOX675	*erm-1(mib10[erm-1[p.T544A]) I; nrfl-1(mib104[nrfl-1a(Δeb = c.1318_1401del)]) IV*
BOX676	*erm-1(mib9[erm-1[p.T544D]) I; nrfl-1(mib104[nrfl-1a(Δeb = c.1318_1401del)]) IV*
BOX677	*nrfl-1(mib104[nrfl-1a(Δeb = c.1318_1401del)]) IV; caIs107[Pges-1::YFP::act-5]*
BOX678	*erm-1(mib10[erm-1[p.T544A]) I; nrfl-1(mib104[nrfl-1a(Δeb = c.1318_1401del)]) IV; caIs107[Pges-1::YFP::act-5]*
BOX679	*erm-1(mib9[erm-1[p.T544D]) I; nrfl-1(mib104[nrfl-1a(Δeb = c.1318_1401del)]) IV; caIs107[Pges-1::YFP::act-5]*

### Cloning and Strain Generation for the SIMPL System

Bait and prey SIMPL constructs were generated using the SapI-based cloning strategy, as previously described (Yao et al., 2020). For the conventional SIMPL system, previously described intein inserts were used (Yao et al., 2020). For the SIMPL-mVenus system, the InteinC-3xFLAG-VC155 and VN155-HA-V5-InteinN inserts were codon-optimized for *C. elegans*, flanked by SapI sites and ordered as gBlocks (IDT). Primers containing the appropriate SapI overhangs were used to amplify *erm-1*, *nrfl-1* and *nrfl-1(Δeb)* from a cDNA library, InteinC-3xFLAG-VC155 from the ordered gBlock and mKate from pDD375 (Addgene #91825). All gBlocks and PCR products were blunt-end cloned into the plasmid pHSG298. Bait or prey, intein, the *rps-0* promoter and the *unc-54* 3’ UTR fragments were combined and inserted into the pMLS257 plasmid (Addgene #73716) using the SapTrap assembly method (Schwartz and Jorgensen, 2016; Yao et al., 2020). Finally for the SIMPL-mVenus system, an mKate2 sequence was integrated into the newly generated *Prps-0::erm-1::VN155-HA-V5-InteinN::unc-54* plasmid. The mKate2 sequence and the *Prps-0::erm-1:VN155-HA-V5-InteinN::unc-54* plasmid were amplified using primers with the appropriate overhangs to incorporate the mKate2 into the plasmid between the promotor and *erm-1* coding sequence using Gibson Assembly (GA). Constructs were verified by Sanger sequencing before injection (Macrogen Europe). Plasmids used for injection were purified using the PureLink HQ Mini Plasmid DNA Purification Kit (Thermo Fisher) using the extra wash step and buffer recommended for endA + strains. Final plasmid sequences are available in Genbank format in Supplementary File S1.

Transgenic animals expressing bait and prey constructs were generated by microinjection in the gonads of young adult N2 animals using an inverted microinjection setup (Eppendorf) with 20 ng/μL of bait and prey plasmids, as well as the pDD382 plasmid (Addgene #91830) containing a visible dominant Rol marker and an hygromycin selection cassette. The DNA mix was spun at max speed on a tabletop centrifuge for 15 min prior to injection. Injected animals were incubated for 2–3 days at 20°C before addition of hygromycin B (250 μg/ml) to the plates. After 1–2 days, surviving Rol animals were singled, allowed to develop, and F2 progeny was screened for successful transmission of the transgenic extrachromosomal array. Multiple lines with successful transmission were saved and used for analysis.

### Western Blot SIMPL Analysis

Animals were grown on NGM plates supplemented with hygromycin B (250 μg/ml) until plates were full, washed off with M9 buffer (0.22 M KH_2_PO, 0.42 M Na_2_HPO_4_, 0.85 M NaCl, 0.001 M MgSO_4_), washed three times with M9 buffer, and incubated at room temperature (RT) for 20 min. Samples were then pelleted and resuspended in 100–200 µL of lysis buffer (25 mM Tris-HCl pH 7.5, 150 mM NaCl, 1 mM EDTA, 0.5% IGEPAL CA-630 (Sigma-Aldrich), 1 tablet/50 ml cOmplete protease inhibitor cocktail (Sigma-Aldrich)), and sonicated with a Diagenode BioRupter Plus for 10 min with the high setting and on/off cycles of 30 s in a 4 °C water bath. The lysates were spun at max speed for 15 min, an equal volume of 2 × SDS buffer (100 mM Tris-HCl, 4% SDS, 0.2% bromophenol blue, 20% glycerol, and 10% β-mercaptoethanol) was added, and boiled 10 min. Depending on the experiment, 5–12 µL of protein lysate was loaded into pre-cast protein gels (4–12% Bolt Bis Tris Plus, ThermoFisher) together with 10 µL of the molecular marker (PageRuler prestained, ThermoFisher). Gels were run for 30–45 min at 200 V in NuPAGE MOPS SDS Running buffer (ThermoFisher), and transferred onto a PVDF membrane (Immobilon-P 0.45 µm, Millipore) at 4°C and 30 V overnight in Bolt transfer buffer (Thermo Fisher). For staining, membranes were rinsed in TBST (50 mM Tris-Cl, 150 mM NaCl, 0.1% Tween-20), blocked with 4% milk in TBST for 1 h at RT, and incubated with primary antibodies in milk for 1 h at RT. Membranes were washed three times for 10 min in TBST, incubated with secondary antibodies in milk for 1 h at RT, and washed again three times for 10 min in TBST before exposure using ECL (SignalFire Plus, Cell signaling). The following antibodies and concentrations were used: rabbit anti-V5, 1:1000 (Cell Signaling #13202); mouse anti-FLAG, 1:10000 (Sigma #F1804); goat anti-Rabbit and donkey anti-mouse HRP conjugates, 1:5000.

### CRISPR/Cas9 Genome Engineering

The *nrfl-1::mCherry*, *nrfl-1(Δeb)* and *nrfl-1(Δeb)::mCherry* strains were engineered by homology-directed repair of CRISPR/Cas9-induced DNA double-strand breaks (DSBs), while the *nrfl-1(null)* deletion was generated by imprecise repair of CRISPR/Cas9-induced DSBs. Delivery of components for CRISPR/Cas9 editing was done by microinjection in the gonads of young adult animals of different genetic backgrounds: *nrfl-1(Δeb)* and *nrfl-1(mib59)* were generated in an N2 background; *nrfl-1::mCherry* was generated in BOX273 background, and *nrfl-1(Δeb)::mCherry* in a BOX422 background. All sequences of the oligonucleotides and crRNAs used (synthesized by IDT) are listed in Table 2.

**TABLE 2:** List of DNA and RNA sequences used

*SIMPL system*	
erm-1 SapI forward	CTG​CTC​TTC​GAA​GAT​GTC​GAA​AAA​AGC​GAT​CAA
erm-1 SapI reverse	CTG​CTC​TTC​GCG​TCA​TAT​TTT​CGT​ATT​GAT​CGA
nrfl-1 SapI forward	CTG​CTC​TTC​GAA​GAT​GGT​GCA​CAT​TCC​GAG​CGA
nrfl-1 SapI reverse	CTG​CTC​TTC​GCG​TCA​TGT​TGC​TGA​CCA​ATT​GAT
nrfl-1(Δeb) SapI reverse	AGGCTCTTCGCGTAGCTTCTCTTGCTGACAFAAT
InteinC-3xFLAG-VC155 SapI forward	GAG​CTC​TTC​GAC​GAT​GGA​CGA​GCG​TGA​GCT​TA
InteinC-3xFLAG-VC155 SapI reverse	GAG​CTG​CTC​TTC​GGC​ACT​TGT​AGA​GCT​CAT​CCA​TTC
InteinC-3xFLAG-VC155 GA forward	TCG​GAC​ACC​GTA​TGT​CGA​AAA​AAG​CGA​TC
InteinC-3xFLAG-VC155 GA reverse	TCG​GAG​ACC​ATA​TTA​CCT​TAA​AAT​TCA​AAA​ATT​AAT​TTC​AG
mKate2 GA forward	TTT​TAA​GGT​AAT​ATG​GTC​TCC​GAG​CTC​ATT​AAA​GAA​AAC
mKate2 GA reverse	TTT​TTT​CGA​CAT​ACG​GTG​TCC​GAG​CTT​GGA​TG
*nrfl-1(null)*	
nrfl-1 sgRNA 5′ forward oligo 1	TCT​TGT​CGC​TCG​GAA​TGT​GCA​CCA
nrfl-1 sgRNA 5′ reverse oligo 1	AAA​CTG​GTG​CAC​ATT​CCG​AGC​GAC
nrfl-1 sgRNA 5′ forward oligo 2	TCT​TGT​CAA​CGA​CAC​AAA​GTC​TTG​G
nrfl-1 sgRNA 5′ reverse oligo 2	AAA​CCC​AAG​ACT​TTG​TGT​CGT​TGA​C
nrfl-1 sgRNA 3′ forward oligo 1	TCT​TGC​CTT​AAC​GAG​AAG​TAT​CAA​T
nrfl-1 sgRNA 3′ reverse oligo 1	AAA​CAT​TGA​TAC​TTC​TCG​TTA​AGG​C
nrfl-1 sgRNA 3′ forward oligo 2	TCT​TGC​CAA​TTG​ATA​CTT​CTC​GTT​A
nrfl-1 sgRNA 3′ reverse oligo 2	AAA​CTA​ACG​AGA​AGT​ATC​AAT​TGG​C
Deletion forward primer	TGG​ACA​GTT​CGT​TGG​TAC​CG
Deletion reverse primer	TAC​ACG​CGC​AAA​GTG​ACC​TA
*nrfl-1(Δeb)*	
nrfl-1 EB sgRNA 5′	UUU​AAU​CUU​CAU​GCU​GAA​CG
nrfl-1 EB sgRNA 3′	AUU​GAU​ACU​UCU​CGU​UAA​GG
ssODN repair template	ACG​ATG​ATA​TCT​ATC​ATT​TGT​CAG​CAA​GAG​AAG​CTA​CGA​TGA​TAT​CTA​TCA​TTT​GTC​AGC​AAG​AGA​AGC​T
Integration forward primer	ATG​CAT​CAC​CTC​GAG​GCT​G
Integration reverse primer	TGA​GCG​ATT​GTG​AAA​TGG​AAG​G
*nrfl-1::mcherry* - Combined with both nrfl-1 sgRNAs 3′ of *nrfl-1(null)*	
LH arm forward primer	ACG​TTG​TAA​AAC​GAC​GGC​CAG​TCG​CCG​GCA​TTT​AAT​GCG​CAT​TGG​TCT​GC
LH arm reverse primer step 1	GAC​TAA​TTG​ATA​CTT​CTC​GTT​AAG​ACT​CAT​CTC​GTG​CCT​ACA​ATT
LH arm reverse primer step 2	CCT​GAG​GCT​CCC​GAT​GCT​CCC​ATG​TTG​CTG​ACT​AAT​TGA​TAC​TTC​TCG​T
RH arm forward primer	AGG​ATG​ACG​ATG​ACA​AGA​GAT​AAT​CTT​TTG​CAA​CTT​CTT​CTT​ATT​TTC​TTC
RH arm reverse primer	GGA​AAC​AGC​TAT​GAC​CAT​GTT​ATC​GAT​TTC​ACC​TTC​CAA​TGT​CAG​GTT​CCC
Integration forward primer	TCA​GGG​AGC​CGG​ATC​TGA​TT
Integration reverse primer	CGG​CTG​AAC​AAA​AGG​AGC​AG
*nrfl-1(Δeb)::mcherry–*Combined with nrfl-1 EB sgRNA 5′ of *nrfl-1(Δeb)*	
nrfl-1 EB mCherry sgRNA	TCA​TAA​CAT​TGC​ATA​TTC​AT
ssODN repair template	CCC​CAG​ATC​AAG​AAT​TTG​GTT​TTA​ATC​TTC​ATG​CTG​TTG​ATA​AGT​ATC​ATA​AAG​ATC​ATA​ACA​TTG​CTT​ACA​GCT​GGG​ATA​ATG​TTG​AAA​GAG​TTG​ATA​CTC​GTC​CA
Integration forward primer	GAT​TTG​GCG​GGT​TTT​CGA​GG
Integration reverse primer	CGG​CTG​AAC​AAA​AGG​AGC​AG

For the *nrfl-1::mCherry*, two plasmid-based sgRNAs were used, generated by ligation of annealed oligo pairs into the *pU6::sgRNA* expression vector pJJR50 (Addgene #75026) as previously described (Waaijers et al., 2016). To generate the *nrfl-1::mCherry* repair template we created a custom SEC vector, pJJR83 (Addgene #75028), by replacing a fragment of pDD282 (Addgene #66823) containing the GFP sequence with a similar fragment containing a codon optimized mCherry sequence with synthetic introns using the flanking Bsu36I and BglII restriction sites. Homology arms of about ±750 bp, flanking the DSB site, were amplified from genomic DNA and introduced into pJJR83 as previously described (Dickinson et al., 2015). The sgRNA (100 ng/μL) and SEC repair template (20 ng/μL) plasmids combined with *Peft-3::Cas9* (60 ng/μL; Addgene #46168) and *Pmyo-2::mCherry* co-injection marker (2.5 ng/μL; pCFJ90, Addgene #19327) were micro-injected in the gonad of young adults. Two injected animals were pooled per plate, incubated for 3 days at 20°C, 500 μL of 5 mg/ml hygromycin was added per plate, and non-transgenic Rol animals were selected after 4–5 days. These selected animals were lysed and genotyped with primers flanking the homology arms and confirmed by Sanger sequencing. To eliminate the SEC selection cassette L1 progeny of homozygous Rol animals was heat shocked in a water-bath at 34°C for 1 h.

To generate the *nrfl-1(null)* deletion allele a mix containing *Peft-3::Cas9* (Addgene #46168; 50 ng/μL), two pairs of sgRNA plasmids targeting the 5′ or 3′ ends of the *nrfl-1* open reading frame (75 ng/μL each), and a *dpy-10* sgRNA plasmid (50 ng/μL) for co-CRISPR selection (Arribere et al., 2014) were micro-injected in the gonad of young adults. To select for deletions, injected animals were transferred to individual plates, incubated for 3–4 days at 20°C, and 96 non-transgenic F1 animals (wild-type, Dpy, or Rol) from 2–3 plates containing high numbers of Dpy and Rol animals were selected and transferred to individual plates. After laying eggs, F1 animals were lysed and genotyped with primers flanking the *nrfl-1* ORF. In all cases, deletions were confirmed by Sanger sequencing. Sanger sequencing was also used to determine the precise molecular lesion in selected animals. The *nrfl-1(null)* allele used in this paper, *nrfl-1(mib59)*, consists of a 9 bp deletion starting 1 bp before the initial base of the start codon of long *nrfl-1* isoforms (a, c, d, h, j), and a second 11,537 bp deletion spanning part of the third exon (791 bp from start of *nrfl-1a*) until the downstream intergenic region, which includes the entire ORFs of the small *nrfl-1* isoforms (left flank 5′- atg​ctt​gtg​atc​tct​gaa​gaa​gga​g, right flank 5′ aat​atc​acg​aac​aac​ttc​tag​gag​c). The *mib59* allele also deleted an ncRNA (C01F6.16) and three piRNAs (C01F6.10, F32B2.25, and F23B2.28) located within *nrfl-1* introns.

The NRFL-1 EB domain deletions were generated using the Alt-R CRISPR-Cas9 system (IDT). A single-stranded oligodeoxynucleotide with about 35 bp homology arms was used as a repair template to fuse the flanks of a deletion spanning nucleotides 1390–1473 of *nrfl-1h*, as previously described (Dokshin et al., 2018). A mix of 250 ng/μL Cas9 protein, 2 μM repair template, 4.5 μM each *nrfl-1* crRNAs, 10 μM tracrRNA, as well as 1 μM *dpy-10* crRNA and ssODN repair for co-CRISPR selection (Arribere et al., 2014) was micro-injected into the gonads of young adults. Animals were selected as described above for the *nrfl-1(null)* allele and genotyped using two primers flanking the deletion.

### Microscopy and Image Analysis

Imaging of *C. elegans* was done by mounting embryos or larvae on a 5% agarose pad in 20 mM Tetramisole solution in M9 to induce paralysis. Spinning disk confocal imaging was performed using a Nikon Eclipse Ti-U manual microscope equipped with a Yokogawa CSU-X1 spinning disk using a 60× 1.4 NA objective, 488 and 561 nm lasers, Semrock 488 long-pass, 525/30 (green), 617/73 (red) & 512/630 (dual) emission filters, 600 Texas Red (EX540-580/DM595/BA600-660) filter blocks, and Andor iXON DU-885 camera. Imaging for FRAP and immunohistochemistry experiments was performed on a Nikon Eclipse-Ti with Perfect Focus System microscope equipped with a Yokogawa CSU-X1-A1 spinning disk using 60× and 100× 1.4 NA objectives, Chroma ET-DAPI (49000), ET-GFP (49002), ET-mCherry (49008) emission filters, 355 nm, 488 nm, 491 nm, and 561 nm lasers, and a Photometrics Evolve 512 EMCCD camera. Targeted photobleaching was done using an ILas system (Roper Scientific France/PICT-IBiSA, Institut Curie). Spinning disk images were acquired using MetaMorph Microscopy Automation and Image Analysis Software. All stacks along the z-axis were obtained at 0.25 μm intervals. Super resolution images of the microvilli were obtained using a Zeiss AxioObserver 7 SP microscope with Definite Focus 2 operated by Zeiss ZEN software with an Airyscan 32-channel GaAsP-PMT area detector using a 100× 1.46 NA objective, and Laser Argon Multiline and 561 nm lasers. Maximum intensity Z projections were done in ImageJ (Fiji) software (Schindelin et al., 2012; Rueden et al., 2017). For quantifications, the same laser power and exposure times were used within experiments. Image scales were calibrated for each microscope using a micrometer slide. For display in figures, level adjustments, false coloring, and image overlays were done in Adobe Photoshop. Image rotation, cropping, and panel assembly were done in Adobe Illustrator. All edits were done non-destructively using adjustment layers and clipping masks, and images were kept in their original capture bit depth until final export from Illustrator for publication.

### Quantitative Image Analysis

Quantitative analysis of spinning disk images was done in Fiji. All values were corrected for background levels by subtracting the average of three regions within the field of view that did not contain any animals. For quantification of apical protein levels, measurements were done in intestinal cells forming int2 through int6, and where the opposing apical membranes could be clearly seen as two lines. Levels were obtained by averaging the peak values of intensity profiles from three 25 px-wide (10 px-wide for the SIMPL-mVenus system) line scans perpendicular to the membrane per animal. For YFP::ACT-5, which is expressed from a transgene with variable expression levels, we express apical enrichment as the ratio of apical/cytoplasmic. Cytoplasmic levels were measured by averaging three regions within the cytoplasm of intestinal cells. Intensity distribution profiles to analyze co-distribution of NRFL-1 with ERM-1 and ACT-5 were obtained by taking three 25 px-wide line scans perpendicular to the apical membrane in each animal. Before averaging these three values, they were aligned and normalized to the peak value. Measurements of multiple animals were again aligned based on the peak value. All presented graphs were made using GraphPad Prism and Adobe Illustrator.

### Protein Degradation

For protein degradation using the anti-GFP-nanobody::ZIF-1 approach (Wang et al., 2017), gonads of young adult BOX428 animals were microinjected with 30 ng/μL *Pelt-2::*α*-GFP-NB::ZIF-1* and 2.5 ng/μL *Pmyo-2::GFP* (#Addgene 26347) as a co-injection marker. Transgenic F1 animals were transferred to individual plates, F2 progeny was screened for successful transmission of the extrachromosomal array and imaged using spinning disk microscopy.

### Brood Size

L4 animals were put on individual plates at 20°C and transferred to a new plate daily until they died. After the parent was removed from a plate, hatched animals and the unhatched eggs were counted 2–4 days later. The number of animals and unhatched eggs combined constitutes the total progeny size. The graph presented was made using GraphPad Prism and Adobe Illustrator.

### Texas Red-Dextran Assay

Mixed stage populations were collected in M9 and washed two times in M9. Animals were then pelleted, concentrated, resuspended in 1 mg/ml Texas Red-dextran 40,000 MW (Thermofisher D1829) in egg buffer (118 mM NaCl, 48 mM KCl, 2 mM MgCl2, 2 mM CaCl2, 25 mM HEPES pH 7.3), and incubated for 60 min on a shaker at 500 rpm. The dye in solution was removed by washing the samples with M9 two times. Animals were paralyzed in 10 mM Tetramisole, transferred to an agarose pad on a glass slide, and imaged using spinning disk microscopy.

### FRAP Experiments and Analysis

For FRAP assays, laser power was adjusted in each experiment to avoid complete photobleaching of the selected area, as the time scale of experiments prevented assessment of photo-induced damage. Photobleaching was performed on a circular region with a diameter of 30 or 40 px at the cortex, and images were taken just before bleaching, directly after, after 15 min, and after 45 min. These images were analyzed using ImageJ. The size of the area for FRAP analysis was defined by the full width at half maximum of an intensity plot across the bleached region. For each time point, the mean intensity value within the bleached region was determined, and the background, defined as the mean intensity of a non-bleached region outside the animal, was subtracted. The mean intensities within the bleached region were corrected for acquisition photobleaching per frame using the background-subtracted mean intensity of a similar non-bleached region at the cortex, which was normalized to the corresponding pre-bleach mean intensity. FRAP recovery was calculated as the change in corrected intensity values within the bleached region from the first image after bleaching normalized to the mean intensity just before bleaching.

### Immunohistochemistry

For the staining of larval stages, embryos were obtained from gravid adults by bleaching and allowed to hatch and develop on plates at 15°C for 24 h. Animals were collected from plates and washed three times with M9 and once with MQ H_2_O before being transferred to poly-L-lysine-coated frosted slides. For the staining of embryos, embryos were obtained from gravid adults by dissection in MQ H_2_O on poly-L-lysine-coated frosted slides and allowed to develop at RT for 4 h. A coverslip (Carl Roth, #1) was lowered on top of larvae/embryos, followed by freezing in liquid nitrogen and snapping off of the coverslip. Fixation was performed in formaldehyde solution with phosphatase inhibitors (3,7% formaldehyde (Sigma-Aldrich), 250 µM EDTA and 50 mM NaF in PBS (1,35 M NaCl, 27 mM KCl, 100 mM Na_2_HPO_4_, 18 mM KH_2_PO_4_)) at RT for 10 min. Samples were rinsed in PBS, permeabilized (PBS + 0,5% triton X-100 (Sigma-Aldrich)) for 30 min, washed four times in wash buffer (0,1% Triton X-100, 250 µM EDTA and 50 mM NaF in PBS) for 10 min each and then blocked (1% bovine serum albumin (Sigma-Aldrich) and 10% goat serum (Sigma-Aldrich)) for 1 h at RT. For the staining with protein phosphatase treatment, samples were treated with Lambda Protein phosphatase (NEB) for 30 min at 30°C followed with an additional four times washing step before they were blocked. Primary antibodies (anti-phospho-ezrin (Thr567)/radixin (Thr564)/moesin (Thr558) (48G2) rabbit mAb #3726 (Cell Signaling Technologies) 1:200 and mouse anti-DLG (Hybridoma bank) 1:50) in blocking solution were applied overnight at 4°C. Samples were then washed four times in wash buffer for 10 min each and stained with secondary antibodies (Alexa-Fluor 488 goat anti-rabbit and Alexa-Fluor 568 goat anti-mouse (Life Technologies, A-11008 and A11004), both 1:500) in blocking solution for 1 hour at RT. Samples were then washed four times in wash buffer and once in PBS for 10 min each and finally mounted with Prolong Gold Antifade with DAPI (Thermofisher) under a coverslip and sealed with nail polish.

### Statistical Analysis

All statistical analyses were performed using GraphPad Prism 8. For population comparisons, a D’Agostino and Pearson test of normality was first performed to determine if the data was sampled from a Gaussian distribution. For data drawn from a Gaussian distribution, comparisons between two populations were done using an unpaired *t*-test, with Welch’s correction if the SDs of the populations differed significantly, and comparisons between >2 populations were done using a one-way ANOVA, or a Welch’s ANOVA if the SDs of the populations differed significantly. For data not drawn from a Gaussian distribution, a non-parametric test was used (Mann-Whitney for 2 populations and Kruskal-Wallis for >2 populations). ANOVA and non-parametric tests were followed up with multiple comparison tests of significance (Dunnett’s, Tukey’s, Dunnett’s T3 or Dunn’s). Tests of significance used and sample sizes are indicated in the figure legends. No statistical method was used to pre-determine sample sizes. No samples or animals were excluded from analysis. The experiments were not randomized, and the investigators were not blinded to allocation during experiments and outcome assessment.

## Data Availability

The original contributions presented in the study are included in the article/Supplementary Material, further inquiries can be directed to the corresponding author.

## Supplementary Material

Supplementary Figure S1 NRFL-1 is recruited to the apical domain by ERM-1 in different tissues. (A) Schematic representation of the domain organization of *C. elegans* NRFL-1, *D. melanogaster* Sip1 and *H. sapiens* NHERF1/EBP50 and NHERF2. Percentages above the domains represent the similarity between that domain and the corresponding domain of NRFL-1. For the single PDZ domain of Sip1, two percentages are presented corresponding to each NRFL-1 PDZ domain. In the EB domain alignment, amino acids that are important for the interaction with ERM proteins are shown in red (Terawaki et al., 2006). PDZ = Post-synaptic density-95, disks-large and zonula occludens-1; EB = ERM binding.

Supplementary Figure S2 Lumen discontinuities in *nrfl-1* and *erm-1* single and double mutants. **(A)** Representative images of the intestine in L2 larvae of the indicated genotypes expressing YFP::ACT-5 as an apical marker. **(B)** L1 larvae carrying the apical marker YFP::ACT-5 of the indicated genotypes fed with Texas-Red Dextran. The orange arrowhead indicates the constriction that prevents the flow of fluorescent dye along the intestine. All images are taken using a spinning-disk confocal microscope, and a single focal plane is shown.

Supplementary Figure S3 Validation of the specificity of the α-pERM for T544 phosphorylated ERM-1. **(A,B)** Representative images of fixed animals stained with antibodies recognizing the junctional protein DLG-1 (α-DLG) and phosphorylated ERM-1 (α-pERM). (A) shows *erm-1(+)* and *erm-1[T544A]* larvae and **(B)** shows the intestine of wild-type 2.5-fold embryos that are untreated (-PP) and treated with protein phosphatase (+PP). All images are taken using a spinning-disk confocal microscope, and maximum intensity projections are presented.

## References

[B1] Abbattiscianni A. C. Favia M. Mancini M. T. Cardone R. A. Guerra L. Monterisi S. (2016). Correctors of Mutant CFTR Enhance Subcortical cAMP-PKA Signaling through Modulating Ezrin Phosphorylation and Cytoskeleton Organization. J. Cel Sci. 129, 1128–1140. 10.1242/jcs.177907 26823603

[B2] Arribere J. A. Bell R. T. Fu B. X. H. Artiles K. L. Hartman P. S. Fire A. Z. (2014). Efficient Marker-free Recovery of Custom Genetic Modifications with CRISPR/Cas9 in *Caenorhabditis elegans* . Genetics 198, 837–846. 10.1534/genetics.114.169730 25161212 PMC4224173

[B3] Barret C. Roy C. Montcourrier P. Mangeat P. Niggli V. (2000). Mutagenesis of the Phosphatidylinositol 4,5-Bisphosphate (Pip2) Binding Site in the Nh2-Terminal Domain of Ezrin Correlates with its Altered Cellular Distribution. J. Cel Biol. 151, 1067–1080. 10.1083/jcb.151.5.1067 PMC2174347 11086008

[B4] Bernadskaya Y. Y. Patel F. B. Hsu H.-T. Soto M. C. (2011). Arp2/3 Promotes junction Formation and Maintenance in theCaenorhabditis Elegansintestine by Regulating Membrane Association of Apical Proteins. Mol. Biol. Cell. 22, 2886–2899. 10.1091/mbc.e10-10-0862 21697505 PMC3154884

[B5] Bonilha V. L. Finnemann S. C. Rodriguez-Boulan E. (1999). Ezrin Promotes Morphogenesis of Apical Microvilli and Basal Infoldings in Retinal Pigment Epithelium. J. Cel Biol. 147, 1533–1548. 10.1083/jcb.147.7.1533 PMC2174247 10613910

[B6] Boratkó A. Csortos C. (2013). NHERF2 Is Crucial in ERM Phosphorylation in Pulmonary Endothelial Cells. Cell Commun. Signal. CCS 11, 99. 10.1186/1478-811X-11-99 24364877 PMC3880038

[B7] Brenner S. (1974). The Genetics of Caenorhabditis Elegans. Genetics 77, 71–94. 10.1093/genetics/77.1.71 4366476 PMC1213120

[B8] Broere N. Chen M. Cinar A. Singh A. K. Hillesheim J. Riederer B. (2009). Defective Jejunal and Colonic Salt Absorption and alteredNa+/H+ Exchanger 3 (NHE3) Activity in NHE Regulatory Factor 1 (NHERF1) Adaptor Protein-Deficient Mice. Pflugers Arch. 457, 1079–1091. 10.1007/s00424-008-0579-1 18758809 PMC3746809

[B9] Bryant D. M. Roignot J. Datta A. Overeem A. W. Kim M. Yu W. (2014). A Molecular Switch for the Orientation of Epithelial Cell Polarization. Develop. Cel 31, 171–187. 10.1016/j.devcel.2014.08.027 PMC4248238 25307480

[B10] Coscoy S. Waharte F. Gautreau A. Martin M. Louvard D. Mangeat P. (2002). Molecular Analysis of Microscopic Ezrin Dynamics by Two-Photon FRAP. Proc. Natl. Acad. Sci. 99, 12813–12818. 10.1073/pnas.192084599 12271120 PMC130542

[B11] Dickinson D. J. Pani A. M. Heppert J. K. Higgins C. D. Goldstein B. (2015). Streamlined Genome Engineering with a Self-Excising Drug Selection Cassette. Genetics 200, 1035–1049. 10.1534/genetics.115.178335 26044593 PMC4574250

[B12] Dokshin G. A. Ghanta K. S. Piscopo K. M. Mello C. C. (2018). Robust Genome Editing with Short Single-Stranded and Long, Partially Single-Stranded DNA Donors in *Caenorhabditis elegans* . Genetics 210, 781–787. 10.1534/genetics.118.301532 30213854 PMC6218216

[B13] Fehon R. G. McClatchey A. I. Bretscher A. (2010). Organizing the Cell Cortex: the Role of ERM Proteins. Nat. Rev. Mol. Cel Biol. 11, 276–287. 10.1038/nrm2866 PMC2871950 20308985

[B14] Fievet B. T. Gautreau A. Roy C. Del Maestro L. Mangeat P. Louvard D. (2004). Phosphoinositide Binding and Phosphorylation Act Sequentially in the Activation Mechanism of Ezrin. J. Cel Biol. 164, 653–659. 10.1083/jcb.200307032 PMC2172172 14993232

[B15] Finnerty C. M. Chambers D. Ingraffea J. Faber H. R. Karplus P. A. Bretscher A. (2004). The EBP50-Moesin Interaction Involves a Binding Site Regulated by Direct Masking on the FERM Domain. J. Cel Sci. 117, 1547–1552. 10.1242/jcs.01038 15020681

[B16] Garbett D. LaLonde D. P. Bretscher A. (2010). The Scaffolding Protein EBP50 Regulates Microvillar Assembly in a Phosphorylation-dependent Manner. J. Cel Biol. 191, 397–413. 10.1083/jcb.201004115 PMC2958488 20937695

[B17] Gary R. Bretscher A. (1995). Ezrin Self-Association Involves Binding of an N-Terminal Domain to a Normally Masked C-Terminal Domain that Includes the F-Actin Binding Site. Mol. Biol. Cell. 6, 1061–1075. 10.1091/mbc.6.8.1061 7579708 PMC301263

[B18] Georgescu M.-M. Cote G. Agarwal N. K. White C. L. (2014). NHERF1/EBP50 Controls Morphogenesis of 3D Colonic Glands by Stabilizing PTEN and Ezrin-Radixin-Moesin Proteins at the Apical Membrane. Neoplasia 16, 365–374.e2. 10.1016/j.neo.2014.04.004 24862762 PMC4094837

[B19] Göbel V. Barrett P. L. Hall D. H. Fleming J. T. (2004). Lumen Morphogenesis in *C. elegans* Requires the Membrane-Cytoskeleton Linker Erm-1. Dev. Cel 6, 865–873. 10.1016/j.devcel.2004.05.018 15177034

[B20] Hagiwara K. Nagamori S. Umemura Y. M. Ohgaki R. Tanaka H. Murata D. (2012). NRFL-1, the *C. elegans* NHERF Orthologue, Interacts with Amino Acid Transporter 6 (AAT-6) for Age-dependent Maintenance of AAT-6 on the Membrane. PLoS ONE 7, e43050. 10.1371/journal.pone.0043050 22916205 PMC3419730

[B21] Hao J.-J. Liu Y. Kruhlak M. Debell K. E. Rellahan B. L. Shaw S. (2009). Phospholipase C-Mediated Hydrolysis of PIP2 Releases ERM Proteins from Lymphocyte Membrane. J. Cel Biol. 184, 451–462. 10.1083/jcb.200807047 PMC2646552 19204146

[B22] Harris T. W. Arnaboldi V. Cain S. Chan J. Chen W. J. Cho J. (2020). WormBase: a Modern Model Organism Information Resource. Nucleic Acids Res. 48, D762–D767. 10.1093/nar/gkz920 31642470 PMC7145598

[B23] Hipfner D. R. Keller N. Cohen S. M. (2004). Slik Sterile-20 Kinase Regulates Moesin Activity to Promote Epithelial Integrity during Tissue Growth. Genes Dev. 18, 2243–2248. 10.1101/gad.303304 15371338 PMC517517

[B24] Hughes S. C. Formstecher E. Fehon R. G. (2010). Sip1, theDrosophilaorthologue of EBP50/NHERF1, Functions with the Sterile 20 Family Kinase Slik to Regulate Moesin Activity. J. Cel Sci. 123, 1099–1107. 10.1242/jcs.059469 PMC2844318 20215404

[B25] Ikenouchi J. Hirata M. Yonemura S. Umeda M. (2013). Sphingomyelin Clustering Is Essential for the Formation of Microvilli. J. Cel Sci. 126, 3585–3592. 10.1242/jcs.122325 23690544

[B26] Ingraffea J. Reczek D. Bretscher A. (2002). Distinct Cell Type-specific Expression of Scaffolding Proteins EBP50 and E3KARP: EBP50 Is Generally Expressed with Ezrin in Specific Epithelia, whereas E3KARP Is Not. Eur. J. Cel Biol. 81, 61–68. 10.1078/0171-9335-00218 11893083

[B27] Kodama Y. Hu C.-D. (2010). An Improved Bimolecular Fluorescence Complementation Assay with a High Signal-To-Noise Ratio. BioTechniques 49, 793–805. 10.2144/000113519 21091444

[B28] Koorman T. Klompstra D. van der Voet M. Lemmens I. Ramalho J. J. Nieuwenhuize S. (2016). A Combined Binary Interaction and Phenotypic Map of *C. elegans* Cell Polarity Proteins. Nat. Cel Biol. 18, 337–346. 10.1038/ncb3300 PMC4767559 26780296

[B29] Kreimann E. L. Morales F. C. de Orbeta-Cruz J. Takahashi Y. Adams H. Liu T.-J. (2007). Cortical Stabilization of β-catenin Contributes to NHERF1/EBP50 Tumor Suppressor Function. Oncogene 26, 5290–5299. 10.1038/sj.onc.1210336 17325659

[B30] LaLonde D. P. Bretscher A. (2009). The Scaffold Protein PDZK1 Undergoes a Head-To-Tail Intramolecular Association that Negatively Regulates its Interaction with EBP50. Biochemistry 48, 2261–2271. 10.1021/bi802089k 19173579 PMC2765514

[B31] LaLonde D. P. Garbett D. Bretscher A. (2010). A Regulated Complex of the Scaffolding Proteins PDZK1 and EBP50 with Ezrin Contribute to Microvillar Organization. Mol. Biol. Cell. 21, 1519–1529. 10.1091/mbc.e10-01-0008 20237154 PMC2861611

[B32] Lamprecht G. Weinman E. J. Yun C.-H. C. (1998). The Role of NHERF and E3KARP in the cAMP-Mediated Inhibition of NHE3. J. Biol. Chem. 273, 29972–29978. 10.1074/jbc.273.45.29972 9792717

[B33] Lazar C. S. Cresson C. M. Lauffenburger D. A. Gill G. N. (2004). The Na+/H+Exchanger Regulatory Factor Stabilizes Epidermal Growth Factor Receptors at the Cell Surface. Mol. Biol. Cell. 15, 5470–5480. 10.1091/mbc.e04-03-0239 15469991 PMC532026

[B34] Li Q. Nance M. R. Kulikauskas R. Nyberg K. Fehon R. Karplus P. A. (2007). Self-masking in an Intact ERM-merlin Protein: An Active Role for the Central α-Helical Domain. J. Mol. Biol. 365, 1446–1459. 10.1016/j.jmb.2006.10.075 17134719 PMC1796844

[B35] Magendantz M. Henry M. D. Lander A. Solomon F. (1995). Interdomain Interactions of Radixin *In Vitro* . J. Biol. Chem. 270, 25324–25327. 10.1074/jbc.270.43.25324 7592691

[B36] Maudsley S. Zamah A. M. Rahman N. Blitzer J. T. Luttrell L. M. Lefkowitz R. J. (2000). Platelet-Derived Growth Factor Receptor Association with Na + /H + Exchanger Regulatory Factor Potentiates Receptor Activity. Mol. Cel. Biol. 20, 8352–8363. 10.1128/mcb.20.22.8352-8363.2000 PMC102142 11046132

[B37] McClatchey A. I. (2014). ERM Proteins at a Glance. J. Cel Sci. 127, 3199–3204. 10.1242/jcs.098343 PMC4117225 24951115

[B38] Morales F. C. Takahashi Y. Kreimann E. L. Georgescu M.-M. (2004). Ezrin-radixin-moesin (ERM)-binding Phosphoprotein 50 Organizes ERM Proteins at the Apical Membrane of Polarized Epithelia. Proc. Natl. Acad. Sci. 101, 17705–17710. 10.1073/pnas.0407974101 15591354 PMC539771

[B39] Na K. Shin H. Cho J.-Y. Jung S. H. Lim J. Lim J.-S. (2017). Systematic Proteogenomic Approach to Exploring a Novel Function for NHERF1 in Human Reproductive Disorder: Lessons for Exploring Missing Proteins. J. Proteome Res. 16, 4455–4467. 10.1021/acs.jproteome.7b00146 28960081 PMC6610236

[B40] Nakamura F. Huang L. Pestonjamasp K. Luna E. J. Furthmayr H. (1999). Regulation of F-Actin Binding to Platelet Moesin *In Vitro* by Both Phosphorylation of Threonine 558 and Polyphosphatidylinositides. Mol. Biol. Cell. 10, 2669–2685. 10.1091/mbc.10.8.2669 10436021 PMC25498

[B41] Oh Y.-S. Heo K. Kim E.-K. Jang J.-H. Bae S. S. Park J. B. (2017). Dynamic Relocalization of NHERF1 Mediates Chemotactic Migration of Ovarian Cancer Cells toward Lysophosphatidic Acid Stimulation. Exp. Mol. Med. 49, e351. 10.1038/emm.2017.88 28684865 PMC5565956

[B42] Pearson M. A. Reczek D. Bretscher A. Karplus P. A. (2000). Structure of the ERM Protein Moesin Reveals the FERM Domain Fold Masked by an Extended Actin Binding Tail Domain. Cell 101, 259–270. 10.1016/s0092-8674(00)80836-3 10847681

[B43] Pelaseyed T. Viswanatha R. Sauvanet C. Filter J. J. Goldberg M. L. Bretscher A. (2017). Ezrin Activation by LOK Phosphorylation Involves a PIP2-dependent Wedge Mechanism. eLife 6, e22759. 10.7554/eLife.22759 28430576 PMC5400502

[B44] Ramalho J. J. Sepers J. J. Nicolle O. Schmidt R. Cravo J. Michaux G. (2020). C-terminal Phosphorylation Modulates ERM-1 Localization and Dynamics to Control Cortical Actin Organization and Support Lumen Formation during *Caenorhabditis elegans* Development. Development 147, dev188011. 10.1242/dev.188011 32586975 PMC10755404

[B45] Reczek D. Berryman M. Bretscher A. (1997). Identification of EBP50: A PDZ-Containing Phosphoprotein that Associates with Members of the Ezrin-Radixin-Moesin Family. J. Cel Biol. 139, 169–179. 10.1083/jcb.139.1.169 PMC2139813 9314537

[B46] Reczek D. Bretscher A. (1998). The Carboxyl-Terminal Region of EBP50 Binds to a Site in the Amino-Terminal Domain of Ezrin that Is Masked in the Dormant Molecule. J. Biol. Chem. 273, 18452–18458. 10.1074/jbc.273.29.18452 9660814

[B47] Roch F. Polesello C. Roubinet C. Martin M. Roy C. Valenti P. (2010). Differential Roles of PtdIns(4,5)P2 and Phosphorylation in Moesin Activation duringDrosophiladevelopment. J. Cel Sci. 123, 2058–2067. 10.1242/jcs.064550 20519583

[B48] Rueden C. T. Schindelin J. Hiner M. C. DeZonia B. E. Walter A. E. Arena E. T. (2017). ImageJ2: ImageJ for the Next Generation of Scientific Image Data. BMC Bioinformatics 18, 529. 10.1186/s12859-017-1934-z 29187165 PMC5708080

[B49] Saotome I. Curto M. McClatchey A. I. (2004). Ezrin Is Essential for Epithelial Organization and Villus Morphogenesis in the Developing Intestine. Develop. Cel 6, 855–864. 10.1016/j.devcel.2004.05.007 15177033

[B50] Schindelin J. Arganda-Carreras I. Frise E. Kaynig V. Longair M. Pietzsch T. (2012). Fiji: an Open-Source Platform for Biological-Image Analysis. Nat. Methods 9, 676–682. 10.1038/nmeth.2019 22743772 PMC3855844

[B51] Schwartz M. L. Jorgensen E. M. (2016). SapTrap, a Toolkit for High-Throughput CRISPR/Cas9 Gene Modification in *Caenorhabditis elegans* . Genetics 202, 1277–1288. 10.1534/genetics.115.184275 26837755 PMC4905529

[B52] Seidler U. Singh A. K. Cinar A. Chen M. Hillesheim J. Hogema B. (2009). The Role of the NHERF Family of PDZ Scaffolding Proteins in the Regulation of Salt and Water Transport. Ann. N. Y. Acad. Sci. 1165, 249–260. 10.1111/j.1749-6632.2009.04046.x 19538313

[B53] Simons P. C. Pietromonaco S. F. Reczek D. Bretscher A. Elias L. (1998). C-terminal Threonine Phosphorylation Activates ERM Proteins to Link the Cell's Cortical Lipid Bilayer to the Cytoskeleton. Biochem. Biophysical Res. Commun. 253, 561–565. 10.1006/bbrc.1998.9823 9918767

[B54] Speck O. Hughes S. C. Noren N. K. Kulikauskas R. M. Fehon R. G. (2003). Moesin Functions Antagonistically to the Rho Pathway to Maintain Epithelial Integrity. Nature 421, 83–87. 10.1038/nature01295 12511959

[B55] Terawaki S.-i. Maesaki R. Hakoshima T. (2006). Structural Basis for NHERF Recognition by ERM Proteins. Structure 14, 777–789. 10.1016/j.str.2006.01.015 16615918

[B56] Van Fürden D. Johnson K. Segbert C. Bossinger O. (2004). The *C. elegans* Ezrin-Radixin-Moesin Protein ERM-1 Is Necessary for Apical junction Remodelling and Tubulogenesis in the Intestine. Dev. Biol. 272, 262–276. 10.1016/j.ydbio.2004.05.012 15242805

[B57] Viswanatha R. Ohouo P. Y. Smolka M. B. Bretscher A. (2012). Local Phosphocycling Mediated by LOK/SLK Restricts Ezrin Function to the Apical Aspect of Epithelial Cells. J. Cel Biol. 199, 969–984. 10.1083/jcb.201207047 PMC3518218 23209304

[B58] Waaijers S. Muñoz J. Berends C. Ramalho J. J. Goerdayal S. S. Low T. Y. (2016). A Tissue-specific Protein Purification Approach in *Caenorhabditis elegans* Identifies Novel Interaction Partners of DLG-1/Discs Large. BMC Biol. 14, 66. 10.1186/s12915-016-0286-x 27506200 PMC4977824

[B59] Wang S. Tang N. H. Lara-Gonzalez P. Zhao Z. Cheerambathur D. K. Prevo B. (2017). A Toolkit for GFP-Mediated Tissue-specific Protein Degradation in *C. elegans* . Development 144, 2694–2701. 10.1242/dev.150094 28619826 PMC5536931

[B60] Weinman E. J. Steplock D. Shenolikar S. (1993). CAMP-mediated Inhibition of the Renal brush Border Membrane Na+-H+ Exchanger Requires a Dissociable Phosphoprotein Cofactor. J. Clin. Invest. 92, 1781–1786. 10.1172/jci116767 8408631 PMC288340

[B61] Yao Z. Aboualizadeh F. Kroll J. Akula I. Snider J. Lyakisheva A. (2020). Split Intein-Mediated Protein Ligation for Detecting Protein-Protein Interactions and Their Inhibition. Nat. Commun. 11, 2440. 10.1038/s41467-020-16299-1 32415080 PMC7229206

[B62] Yonemura S. Matsui T. Tsukita S. Tsukita S. (2002). Rho-dependent and -independent Activation Mechanisms of Ezrin/radixin/moesin Proteins: an Essential Role for Polyphosphoinositides *In Vivo* . J. Cel Sci. 115, 2569–2580. 10.1242/jcs.115.12.2569 12045227

[B63] Yun C. H. C. Oh S. Zizak M. Steplock D. Tsao S. Tse C.-M. (1997). cAMP-mediated Inhibition of the Epithelial brush Border Na+/H+ Exchanger, NHE3, Requires an Associated Regulatory Protein. Proc. Natl. Acad. Sci. 94, 3010–3015. 10.1073/pnas.94.7.3010 9096337 PMC20313

