# Supplementary figures and images for: ERM-1 Phosphorylation and NRFL-1 Redundantly Control Lumen Formation in the C. elegans Intestine
Source: Front Cell Dev Biol. 2022 Feb 7;10:769862. doi: 10.3389/fcell.2022.769862 (PMC8860247; doi:10.3389/fcell.2022.769862)

Figure S2

A

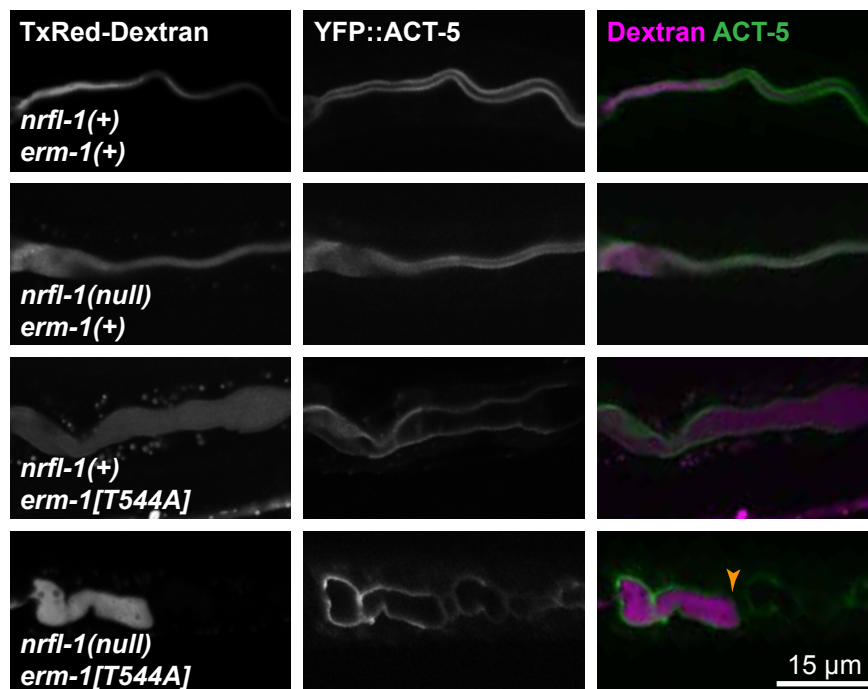

B

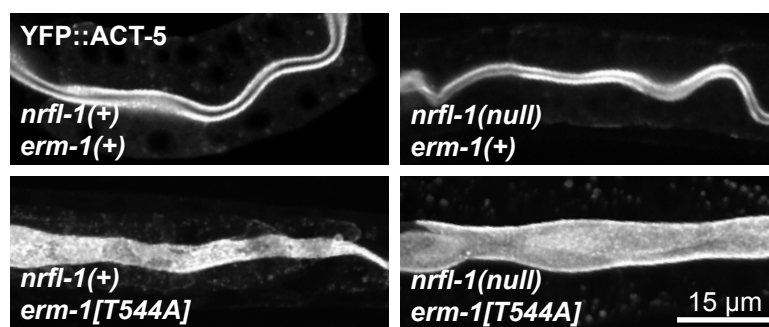

Supplement: Supplementary file 2 [file Image2.pdf]

Figure S3

A

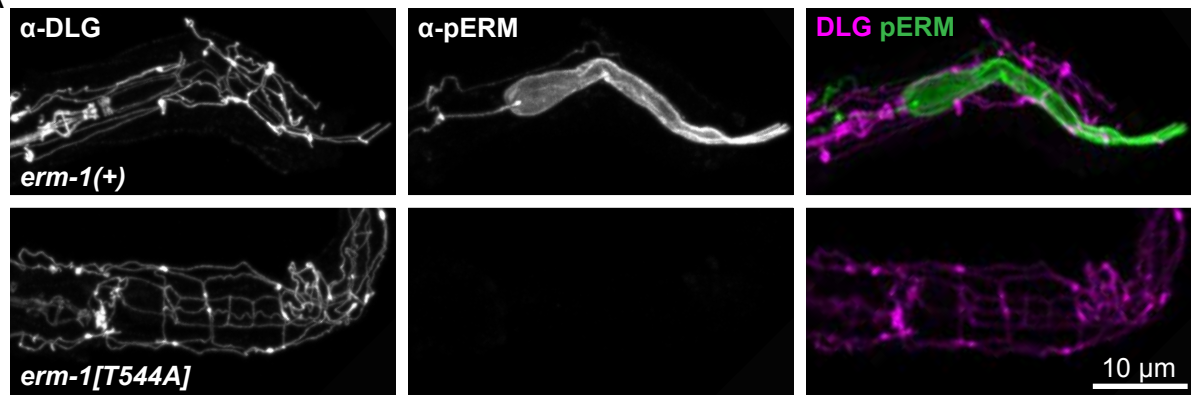

B

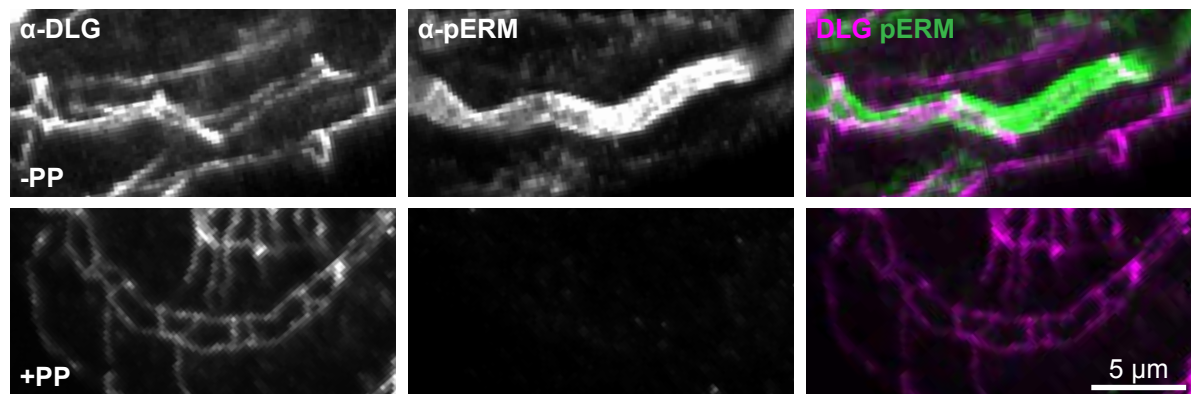

Supplement: Supplementary file 3 [file Image3.pdf]

Figure S1

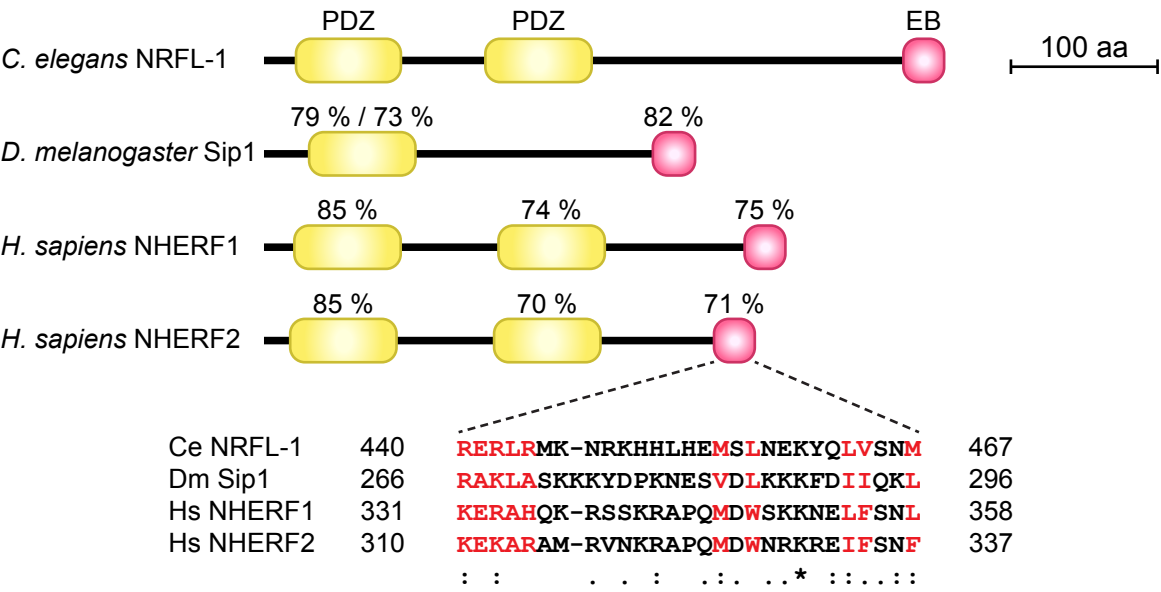

Supplement: Supplementary file 4 [file Image1.pdf]
